# Operando XAS and DFT Uncover Structure-Performance Relationships in Re/TiO_2_ for Selective CO_2_ Hydrogenation to Methanol

**DOI:** 10.1021/acscatal.5c05984

**Published:** 2025-11-04

**Authors:** Maite Lippel Gothe, Adriano Henrique Braga, Lais Reis Borges, Jiyun Hong, Giliandro Farias, Alvaro David Torrez Baptista, Bryan Alberto Laura Larico, Ana Barbara Moulin Cansian, Caetano Rodrigues Miranda, Simon R. Bare, Liane Marcia Rossi, Pedro Vidinha

**Affiliations:** † Departamento de Química Fundamental, 28133 Instituto de Química Universidade de São Paulo, Av Prof Lineu Prestes 748, São Paulo 05508-000, SP, Brazil; ‡ Stanford Synchrotron Radiation Lightsource, 226576 SLAC National Accelerator Laboratory, Menlo Park 94025, California, United States; § Instituto de Física, Universidade de São Paulo, R. do Matão, 1371, São Paulo 05508-090, SP, Brazil

**Keywords:** carbon dioxide, hydrogenation, XAS, rhenium, nanoparticles

## Abstract

The conversion of CO_2_ into value-added chemicals, such as methanol, offers a promising pathway toward a renewable energy future. However, a precise kinetic control and a highly selective catalyst are necessary to overcome the thermodynamic preference for CO_2_ hydrogenation to methane. Rhenium-based catalysts, particularly Re/TiO_2_, demonstrate high activity and selectivity for methanol under high-pressure conditions. For example, at 100 bar and 200 °C, a methanol selectivity of 97–99% was obtained. Catalysts with 1 wt % Re and 5 wt % Re/TiO_2_ were used to study the effect of cluster sizes. At 250 °C, the 1 wt % catalyst achieves 97% selectivity at 23% conversion, whereas 5 wt % Re/TiO_2_ achieves 74% selectivity at 40% conversion, corresponding to a drop in space-time yield from 65 to 16 g_CH_3_OH_·g_Re_^–1^·h^–1^, respectively. X-ray absorption spectroscopy provided insights into the structure of the active sites, while density functional theory calculations revealed the effects of cluster size on the energy barriers for H_2_ activation, CH_3_OH dissociation, and CH_3_OH desorption, all of which directly influence conversion and selectivity. These results underscore the importance of balancing cluster size for optimal catalyst performance and provide insights into the design of efficient and selective catalysts for renewable methanol production.

## Graphical abstract

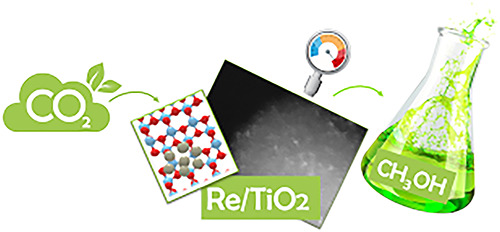

## Introduction

Methanol (CH_3_OH), which can be directly obtained from CO_2_ hydrogenation, is a promising platform molecule, as it can be used as a fuel or integrated into downstream valorization processes to produce gasoline, aviation fuel, or dimethyl ether.^1^ Thermodynamically, CH_4_ is by far the most favorable product of CO_2_ hydrogenation, as its Gibbs energy delta is negative (Δ*G*_298K_ = −113 kJ mol^–1^) while CH_3_OH is not a favored product (Δ*G*_298K_ = 3.5 kJ mol^–1^). The thermodynamic equilibrium constant of the CO_2_ to CH_3_OH reaction (*K*_298K_ = 2.45 × 10^–1^) is 20 orders of magnitude lower than that of CO_2_ to CH_4_ (*K*_298K_ = 7.79 × 10^19^).^2^ Even though high pressure and low temperature favor methanol, the selectivity of the CO_2_ hydrogenation at the thermodynamic equilibrium is >99% selective to CH_4_.^3–5^ Therefore, since the conversion of CO_2_ to methanol must rely on kinetic influences in order to be selective, a suitable catalyst is paramount for the feasibility of this process.^6^

In the hydrogenation of CO_2_ to methanol, Cu, In, and Pd are more commonly identified as active metals in heterogeneous catalysts.^7^ The copper–zinc-alumina mixed oxide catalysts, known as CZA, have been applied on industrial scale processes since the 1940s, and to date, with a reported CO_2_ conversion of 20% and a selectivity of 40%.^8^ A range of transition metals has been explored, with catalysts based on In_2_O_3_ emerging as a highlight.^8–13^ Particularly, a Pd/In_2_O_3_^–^SBA-15 catalyst reached 13% CO_2_ conversion with 84% methanol selectivity.^14^ In hydrogenation catalysis, rhenium has been recognized for its remarkable activity and selectivity in converting carboxylic acids into alcohols, as well as its low susceptibility to catalyst poisoning and deactivation.^15–21^

Rhenium catalysts have also been frequently studied in the hydrogenation of carbonyl and carboxyl groups, demonstrating high selectivity toward the formation of hydroxyl-functionalized products, avoiding overhydrogenation.^22–27^ An efficient supercritical flow process for the hydrogenation of CO_2_ to CH_3_OH over a Re/TiO_2_ catalyst has been reported by our group.^28^ In the high-pressure hydrogenation of CO_2_ to CH_3_OH, the selectivity of rhenium-based catalysts is sensitive to small changes in their synthesis procedure, such as the choice of rhenium precursor or rhenium loading, as these factors can affect the surface composition of the catalyst.^29^ Additionally, recent literature suggests that catalysts composed of subnanometric clusters of rhenium on metal oxide supports present better methanol selectivity than catalysts composed of larger metallic nanoparticles, which lead to methanation.^30–32^

The efficiency and selectivity of CO_2_ hydrogenation to methanol depend on the structural and electronic properties of the catalyst, which can be probed through a combination of experimental and computational techniques.^33,34^ *In situ* and *operando* X-ray absorption spectroscopy (XAS) analyses of the active metal can be particularly helpful in the characterization of the active sites present,^35,36^ and density functional theory (DFT) can provide additional insights to elucidate the catalytic performance of these materials.^37–39^ Here, the supercritical flow process of CO_2_ hydrogenation to CH_3_OH over 1 or 5 wt % Re/TiO_2_ was evaluated over a wide range of reaction conditions. DFT calculations of the dissociation of H_2_ and CH_3_OH on the surface of rhenium-based catalysts, coupled with *operando* XAS data, reveal how the size and structure of rhenium clusters—ranging from subnanometric to larger nanoparticles—affect the energetics and pathways of key reaction steps.

## Experimental Methods

### Materials

Rhenium­(VII) oxide (Re_2_O_7_) was purchased from Sigma-Aldrich at ≥99.9% purity, and titanium oxide P25 was purchased from Degussa at 99.9% purity and 20 nm particle size. Hydrogen gas and carbon dioxide were acquired from Special Gases at 99.5% purity. Carbon monoxide and methane analytical standards were acquired from White Martins at 99.99% purity. The analytical standard for methanol (CH_3_OH) was obtained from Sigma-Aldrich at 99.999% purity. All single-way and double-way valves were purchased from a high-pressure company (HIP, USA). The high-pressure fixed-bed reactor used was produced in-house using stainless steel 316L and Swagelok fittings.

### Catalyst Synthesis and Characterization

The Re/TiO_2_ catalysts were synthesized by the wet impregnation of TiO_2_ (P25, Degussa, 99.9%) with an aqueous solution of 0.1 mg mL^–1^ Re_2_O_7_ (Sigma-Aldrich, ≥99.9%). A suspension of 500 mg of TiO_2_ support to 500 mL of Re_2_O_7_ aqueous solution was left under magnetic stirring overnight, followed by drying at 120 °C in air. Previous to the reactions, the Re/TiO_2_ catalysts were heated under H_2_ in order to reduce the rhenium oxide species to metallic rhenium clusters. The temperature of prereduction is specified for each experiment in the results discussion.

X-ray diffraction (XRD) patterns were obtained with a Rigaku Miniflex diffractometer using Cu Kα radiation, 30 kV tension, and 15 mA current, with a step 2θ of 0.01°. Rhenium percentages on the catalyst were evaluated by Inductively Coupled Plasma Optical Emission Spectrometry (ICP-OES) in a Spectro Arcos spectrometer. Samples were previously digested by heating in a 1:3 mixture of HNO_3_ and HCl. Infrared (IR) spectrometry studies were performed using a Shimadzu IR Prestige 21 spectrometer, which measured spectra obtained from 64 scans at a spectral resolution of 4 cm^–1^. A sample of 20 mg of Re/TiO_2_ was pressed into a pellet and placed into a Specac IR high-temperature transmission cell. The catalyst was pretreated at 500 °C under a flow of 10 mL min^–1^ H_2_ and 50 mL min^–1^ argon (Ar) before measurements. The reaction was studied by flowing 10 mL min^–1^ of CO_2_ and 30 mL min^–1^ of H_2_ in 50 mL min^–1^ of Ar, at ambient pressure and several temperatures.

X-ray photoelectron spectroscopy (XPS) data were obtained with a Specs instrument with monochromatic Al Kα of excitation energy = 1486.71 eV. A constant pass energy of 40 eV and a step of 0.2 eV were applied to all high-resolution spectra, with a dwell time of 0.1 s. The number of scans acquired for Re 4f high-resolution spectra was 50 for the sample with 1 wt % Re and 30 for the sample with 5 wt % Re. For the high-resolution spectra of Ti 2p, 30 scans were sufficient for the analyses of both samples. CasaXPS software was used for peak fitting, and the well-defined Ti (IV) 2p_3/2_ peak was calibrated to 458.6 eV.

X-ray absorption spectroscopy (XAS) data at the Re L_3_-edge (10535 eV) were measured at beamline 2–2 of the Stanford Synchrotron Radiation Lightsource (SSRL). Beamline 2–2 is the center branch of a bent magnet source with a water-cooled double-bounce monochromator equipped with a Si(220) crystal set. The XAS spectra were collected in continuous scanning mode with fluorescence detection using PIPS diode. The spectra of samples with 1 wt % rhenium were collected in a 180 s trajectory, while the spectra of samples with 5 wt % rhenium had a trajectory of 92 s. The samples were packed in a 1/8″ diameter quartz capillary tube as a fixed-bed reactor^40^ to allow for the *in situ* XAS, coupled with product detection by mass spectrometry. The reduction procedure was performed under H_2_ (10% in helium) at a rate of 10 °C min^–1^. The reaction was conducted at a temperature of 200 °C and pressurized to 20 bar with a flow of CO_2_/H_2_ in a 1:4 ratio, and it was followed for 3 h. During the reaction, all XANES were recorded at 200 °C. For steady-state EXAFS, the cell was cooled to room temperature for data collection, and nine scans were averaged to improve the signal-to-noise ratio. The Demeter software package, version 0.9.26, was used for data normalization and EXAFS fitting. For EXAFS modeling, the scattering paths were generated using CIF files from the Inorganic Crystal Structures Database (ICSD). The Re (ICSD-650068) and ReO_2_ (ICSD-24060) files were used to create metallic Re–Re and Re–O paths, respectively.

The H_2_ temperature-programmed reduction (H_2_-TPR) analyses were performed using an AutoChem II 2920 instrument (Micromeritics). A quartz reactor was loaded with 80 mg of catalyst and pretreated at 200 °C under a flow rate of 30 mL min^–1^ of He. The sample was then cooled to room temperature and exposed to a 30 mL min^–1^ flow of 10% H_2_/N_2_ for 20 min, followed by heating to 850 °C at a rate of 10 °C min^–1^. The hydrogen consumption was determined by analysis of the effluent gases using a thermal conductivity detector (TCD). Transmission electron microscopy images were acquired on a JEOL 2100 F or a FEI Talos F200X microscope, operating at an acceleration voltage of 200 kV with a field-emission gun, or FEI Titan Cubed Themis, operating at 300 kV. The images were acquired using conventional TEM and Scanning TEM (STEM) to enhance the contrast between Re and TiO_2_. The catalyst was dispersed in water, and dripped onto an ultrathin carbon film-coated Cu grid (Ted Pella). STEM images were acquired using a high-angle annular dark field detector. A histogram of the nanoparticle size distribution was evaluated through these images by measuring particles and fitting the histogram with a log-normal function.

### Catalytic Tests

The supercritical flow reaction system was built in-house and is described elsewhere.^28^ Briefly, a syringe pump Isco model 500D pressurized the gas mixture of CO_2_ and H_2_ and controlled the flow rate into a fixed-bed reactor (stainless steel tubing, 1/4″, ID: 3.175 mm) packed with 290 mg of the Re/TiO_2_ heterogeneous catalyst. A six-way sampling valve (Rheodyne 7000L) was used to collect samples and evaluate CO_2_ hydrogenation under pressure, and a spring-loaded piston backpressure valve (CITUA, Brazil) controlled the pressure. Analyses of reaction products were performed in a Shimadzu QP2010 gas chromatographer equipped with TCD and MS. CH_4_, CO, and CO2 were analyzed with a Carboxen 1010 column on TCD, and CH_3_OH was analyzed with a Stabilwax column by MS. The high-pressure flow reactions were followed for 6 h. After 1 h of reaction, the product concentration values obtained by GC analyses stabilized, and these were used in the calculation of the conversion, selectivity, and space-time yield values presented here.

### Thermodynamic Equilibrium

The equilibrium composition of the reaction system was determined using the Gibbs free energy minimization method. At equilibrium, the system’s total Gibbs free energy (*G*) reaches its minimum, with its differential equaling zero.^41,42^ Based on this theory, the Aspen Plus software features the RGibbs module, which calculates the composition at equilibrium using only pressure and temperature as input variables, without requiring stochiometric or kinetic information. Version 8.8 of Aspen Plus was utilized to calculate conversion and product selectivities. From the RGibbs input and output compositions, the CO_2_ conversion was calculated using eq 1, the H_2_ conversion using eq 2, and the selectivity using eq 3.

1 $$X_{{\mathrm{CO}}_{2}}=\left[\frac{\left(F_{\mathrm{in},{\mathrm{CO}}_{2}}-F_{\mathrm{out},{\mathrm{CO}}_{2}}\right)}{F_{\mathrm{in},{\mathrm{CO}}_{2}}}\right]\cdot 100$$

2 $$X_{H_{2}}=\left[\frac{\left(F_{\mathrm{in},H_{2}}-F_{\mathrm{out},H_{2}}\right)}{F_{\mathrm{in},H_{2}}}\right]\cdot 100$$

3 $$S_{i}=\left[\frac{\lambda \cdot F_{\mathrm{out},i}}{F_{\mathrm{in},{\mathrm{CO}}_{2}}-F_{\mathrm{out},{\mathrm{CO}}_{2}}}\right]\cdot 100$$

*X_i_*: *i* Conversion; *F*_in,*i*_: Molar flow rate of *i* at inlet; *F*_out,*i*_: Molar flow rate of *i* at outlet; *S_i_*: *i* Selectivity; λ: number of carbon atoms in species *i*.

The SRK equation of state, as modified by Mathias,^43^ was employed in this study, utilizing pure and binary interaction parameters as detailed by Bennekom et al.^44^ Based on the experimentally proposed reaction conditions, the components selected for the simulation were H_2_, CO_2_, CO, CH_3_OH, CH_4_, and H_2_O. Following an initial run at 200 °C and 100 bar, a sensitivity analysis was conducted by varying the temperature (150–300 °C), pressure (1, 20, 40, 60, 80, 100, and 120 bar), and CO_2_/H_2_ ratio (1:1, 1:2, 1:3, 1:4, 1:5, and 1:10). Further details are provided in Supporting Information.

### Theoretical Models and Methodology

Bulk anatase TiO_2_ forms a tetragonal lattice with the space group *I*4_1_/*amd*, and the experimental lattice constants are *a* = *b* = 3.776 Å and *c* = 9.486 Å. We built a periodic slab with four layers for (101) facets. A four-layer 4 × 2 supercell was used. The TiO_2_(101) facets are chosen because anatase TiO_2_(101) facets are the dominant facets in the experimental sample. The bottom two layers of Ti and O are fixed, while the top two are relaxed during the calculation. The vacuum thickness was optimized to be 20 Å.

All calculations were carried out within the DFT formalism in the Vienna Ab Initio Simulation Package (VASP) electronic structure code^45^ using the PBE exchange-correlation functional^46^ and 450 eV plane-wave cutoff. γ-centered *k*-point meshes of 3 × 3 × 1 were used. The PAW method was used to describe the effect of core electrons.^47^ All structures were relaxed until the forces acting on each atom were smaller than 1 × 10^–4^ eV Å^–1^. Transition states along the reaction pathways are searched by the Climbing Image Nudged Elastic Band (CI-NEB) approach.^48,49^ Adsorption energies have been calculated using the formula: $E_{\mathrm{ads}}=E_{\mathrm{adsorbate}+\mathrm{slab}}^{\mathrm{DFT}}-E_{\mathrm{slab}}^{\mathrm{DFT}}-\sum_{i}\alpha_{i}E_{i,\mathrm{gas}}^{\mathrm{DFT}}$ . The free energies of species were calculated as: *G* = *E*_DFT_ + *E*_ZPE_ – *T*·*S*, where *E*_ZPE_ and *T*·*S* of adsorbed species were calculated by vibration analysis.

## Results and Discussion

### Effect of Rhenium wt % and Prereduction under H_2_ on Rhenium Species and Catalytic Activity

The Re/TiO_2_ catalysts were synthesized through wet impregnation of commercially available TiO_2_ (P25) with ReO_4_^–^, derived from an aqueous solution of Re_2_O_7_, followed by drying in air at 120 °C, and a finally a thermal treatment under H_2_ at 250 or 500 °C to produce the metallic rhenium clusters, which is performed in situ in the reactor. However, not all characterization techniques were available with in situ heating under H_2_. The analysis of Re/TiO_2_ with ex situ prereduction could lead to poor interpretation of the results, as rhenium nanoclusters are known to be very easily reoxidized when exposed to air, even if at ambient temperature.^50–52^ Therefore, the as prepared Re/TiO_2_ catalysts were also characterized prior to the reduction, when rhenium oxide species were still present. The understanding of the nanostructure of the rhenium oxides in the as prepared catalysts can aid in the interpretation of the characterizations of in situ reduced metallic Re/TiO_2_. The Raman spectra of the as-prepared Re/TiO_2_ catalysts were measured (Figure 1a) and compared to ReO_2_, ReO_3_, and NH_4_ReO_7_ as standards (Figure S1). Raman measurements were obtained *ex situ* with samples exposed to air, with no prereduction step. As seen on Figure 1a, the sample with 5 wt % Re has a peak similar to that from ReO_3_ around 990 cm^–1^, whereas the catalyst with 1 wt % Re has a peak closer to the NH_4_ReO_4_ standard at 970 cm^–1^, indicating that the as-prepared catalysts have slightly different configurations of supported rhenium oxides.^53^ The presence of peaks in the 970–990 cm^–1^ region of the Raman spectra of Re/TiO_2_ materials is consistent with dioxo or trioxo rhenium species, with multiple Re=O terminal bonds.^54^ Vuurman et al.^55^ also found a 970 cm^–1^ Raman band for a Re_2_O_7_/TiO_2_ as-prepared catalyst, which moved to 1005 cm^–1^ after *in situ* dehydration by thermal treatment at 450 °C under oxygen for 1 h.

**Figure 1:**
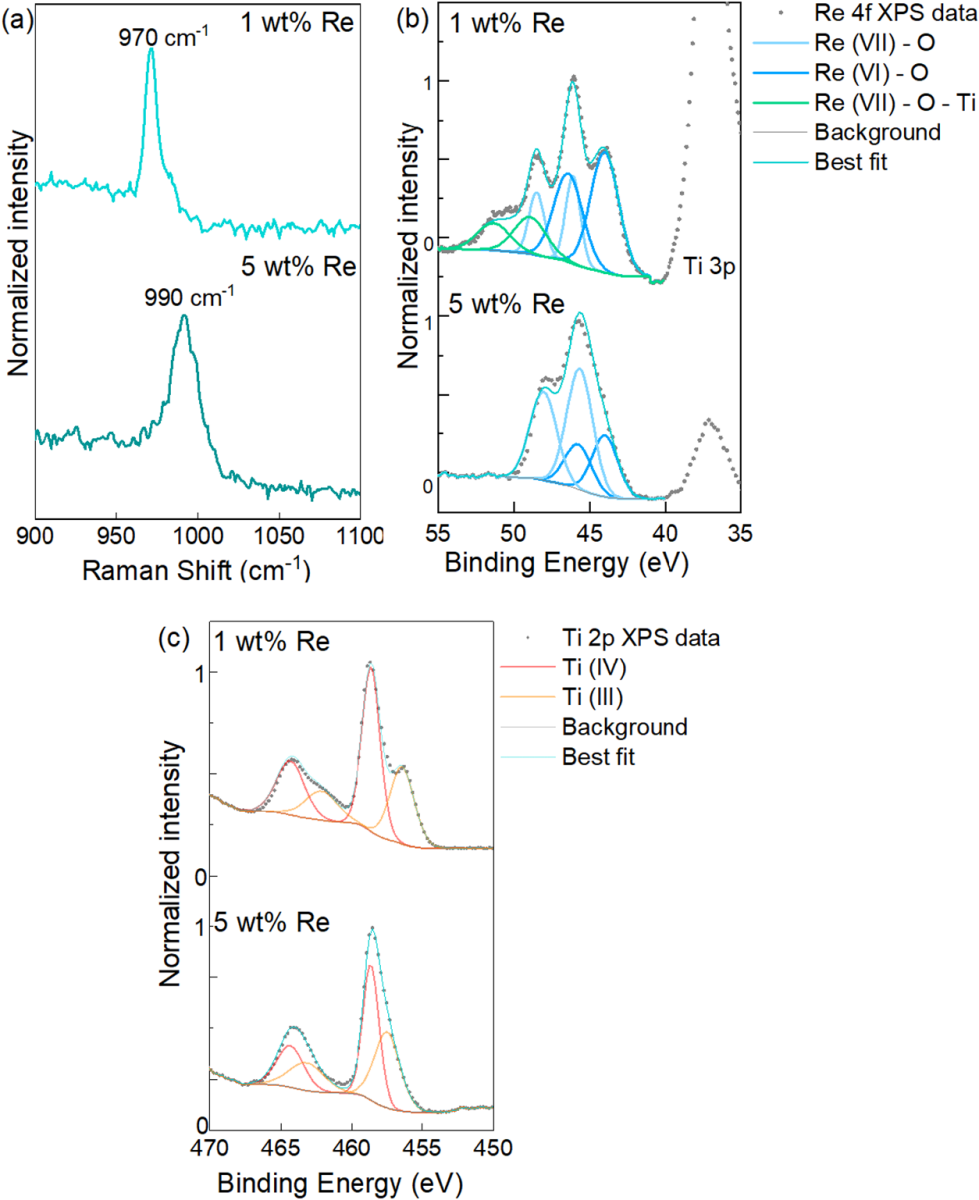
(a) Raman spectra and (b) XPS of rhenium standards and rhenium catalysts Re/TiO_2_ with 5 and 1 wt % Re as prepared in the Re 4f region and (c) Ti 2p region.

The Raman band at 970 cm^–1^ matches that of the symmetric stretching mode of a tetrahedral ReO_4_^–^ in aqueous solution,^56,57^ which is the species present when the aqueous impregnation of TiO_2_ is performed with Re_2_O_7_ as a rhenium precursor, suggesting that rhenium oxide maintains a geometry similar to aqueous Re_2_O_7_^–^ when impregnated on TiO_2_. The higher frequency peak at 990 cm^–1^ relates to symmetric stretching of a terminal Re=O species on adsorbed Re– and can also be observed on ReO_3_ as a surface defect of this oxide.^58^ A shift to higher frequencies of Raman peaks on supported rhenium catalysts has been associated with dehydration of the surface, when comparing the same sample before and after calcination at 450 °C.^55,56^ This could indicate that the 5 wt % Re catalyst contains fewer surface hydroxyls, which is consistent with surface −OH groups serving as anchor sites for Re species upon catalyst synthesis.^59,60^ Raman spectra at their full scale from 200 to 1100 cm^–1^ of these samples can be seen in Figure S1.

XPS analyses of the as-prepared catalysts (Figure 1b and Table 1) suggest that the surface of as-prepared Re/TiO_2_ presents both Re­(VII) and Re­(VI), even though the aqueous impregnation was performed with ReO_4_^–^ which is a Re­(VII) species. At this point, no chemical reduction process had been performed. The presence of Re­(VI) could be due to exposure to X-rays during XPS, which causes the reduction of surface rhenium species.^61,62^ The 5 wt % Re/TiO_2_ sample contains 66 atom % of Re­(VII) at a binding energy of 45.7 eV for the Re 4f_7/2_ component and 34 at% of Re­(VI) at a binding energy of 44.0 eV. The surface of the 1 wt % sample contains 58 atom % of Re­(VI) at 44.0 eV and 22 atom % of Re­(VII) at 46.1 eV. These results are in agreement with the XPS analyses reported for similar Re/TiO_2_ samples.^26,63,64^ An additional component appears in the Re 4f_7/2_ XPS region of 1 wt % Re/TiO_2_ at 48.9 eV, which is over 2 eV higher than the energy levels reported for Re­(VII) in rhenium oxide species,^65^ and accounts for 20% of surface rhenium. Therefore, the presence of a Re 4f_7/2_ peak at 48.9 eV may be related to a Re­(VII) species with strong interaction with the TiO_2_ support in a Re–O–Ti bond with charge transfer from rhenium to titanium,^31,66^ a phenomenon also reported on by Shimizu et al, who demonstrated through experimental and computational approaches that TiO_2_ can accept electrons from rhenium into its conduction band, therefore leading to a strong metal–support interaction.^67^ XPS analyses of the as-prepared catalysts in the Ti 2p region (Figure 1c) show a lower binding energy for Ti­(III) 2p_3/2_ on 1 wt % Re/TiO_2_ (456.4 eV) than on 5 wt % Re/TiO_2_ (457.4 eV), which agrees with the presence of a strong interaction with the support through a Re–O–Ti bond on the sample with lower metal loading.

**Table 1:** XPS Data of the Re 4f_7/2_ Region on As-Prepared Re/TiO_2_ Catalysts

	binding energy (eV)	atom %
5 wt % Re/TiO_2_		
Re(VI)–O	44.0	34
Re(VII)–O	45.7	66
1 wt % Re/TiO_2_		
Re(VI)–O	44.0	58
Re(VII)–O	46.1	22
Re–O–Ti δ+ (∼VII)	48.9	20

The XRD pattern of the as-prepared Re/TiO_2_ catalysts only show peaks due to the TiO_2_ anatase and rutile phases of TiO_2_, consistent with the TiO_2_ P25 used as support, which is a mixture of 80% anatase and 20% rutile. The rhenium oxide species may be too small in size, amorphous, or too low in concentration to be detected by XRD. The 5 wt % Re/TiO_2_ sample prereduced at 500 °C was the only one to show metallic rhenium peaks at 2θ = 37.5° (11̅0), 40.3° (002), 42.8° (11̅1), and 56.3° (11̅2), which correspond to the hexagonal close packed structure of rhenium (ICSD 650068). The XRD data are shown in Figure S2. The HR-STEM analysis of the Re/TiO_2_ catalysts is displayed in Figure 2. The as-prepared 1 wt % Re/TiO_2_ catalyst had no visible rhenium nanoclusters. When reduced at 500 °C, the 1 wt % sample presented subnanometric clusters averaging 0.5 ± 0.2 nm. Meanwhile, on 1 wt % Re/TiO_2_ reduced at a lower temperature of 250 °C, some subnanometric clusters of around 0.2 nm were visible; however, their population was not statistically significant (Figure S3). The 5 wt % Re/TiO_2_ catalyst revealed a uniform distribution of rhenium nanoclusters on the TiO_2_ crystals, with an average size of 1.0 ± 0.2 nm, both on the as-prepared catalyst and when reduced at 250 °C,^28^ with a slight increase to 1.1 ± 0.3 nm on the reduced catalyst at 500 °C under H_2_. The size distribution histograms and fitted curves are shown in Figure S4.

**Figure 2:**
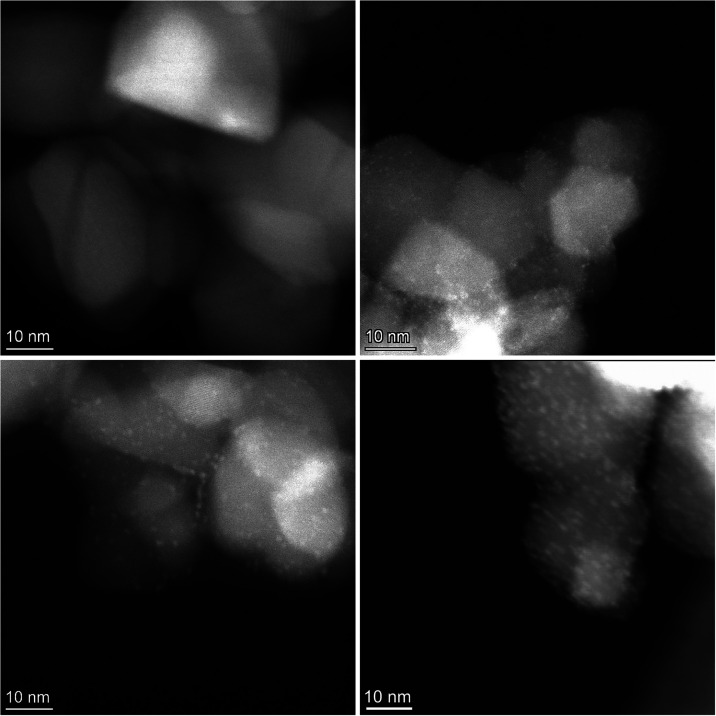
HR-STEM images of as-prepared 1 wt % Re/TiO_2_ (top left), 1 wt % reduced at 500 °C (top right), as-prepared 5 wt % Re/TiO_2_ (bottom left) and 5 wt % reduced at 500 °C (bottom right).

To further elucidate the structure of Re/TiO_2_, a series of XAS experiments were conducted on as-prepared 1 and 5 wt % Re/TiO_2_ (after aqueous impregnation of Re_2_O_7_ on TiO_2_ and air-dried at 120 °C), which were then reduced *in situ* under H_2_ (diluted to 5% in He) from room temperature up to 500 °C. As seen on Re L_3_-edge XANES spectra collected at room temperature (Figure 3a), the spectra of Re/TiO_2_ vary with the weight percentage of rhenium. The absorption edge energy, defined as the maximum of the first derivative of μE plotted against energy, is very similar for the two as-prepared catalysts of different rhenium loading (Figure S5). After reduction at 500 °C, the white line for each catalyst is substantially less intense, and the absorption edge is shifted to a lower value, both of which are consistent with the reduction of rhenium.

**Figure 3:**
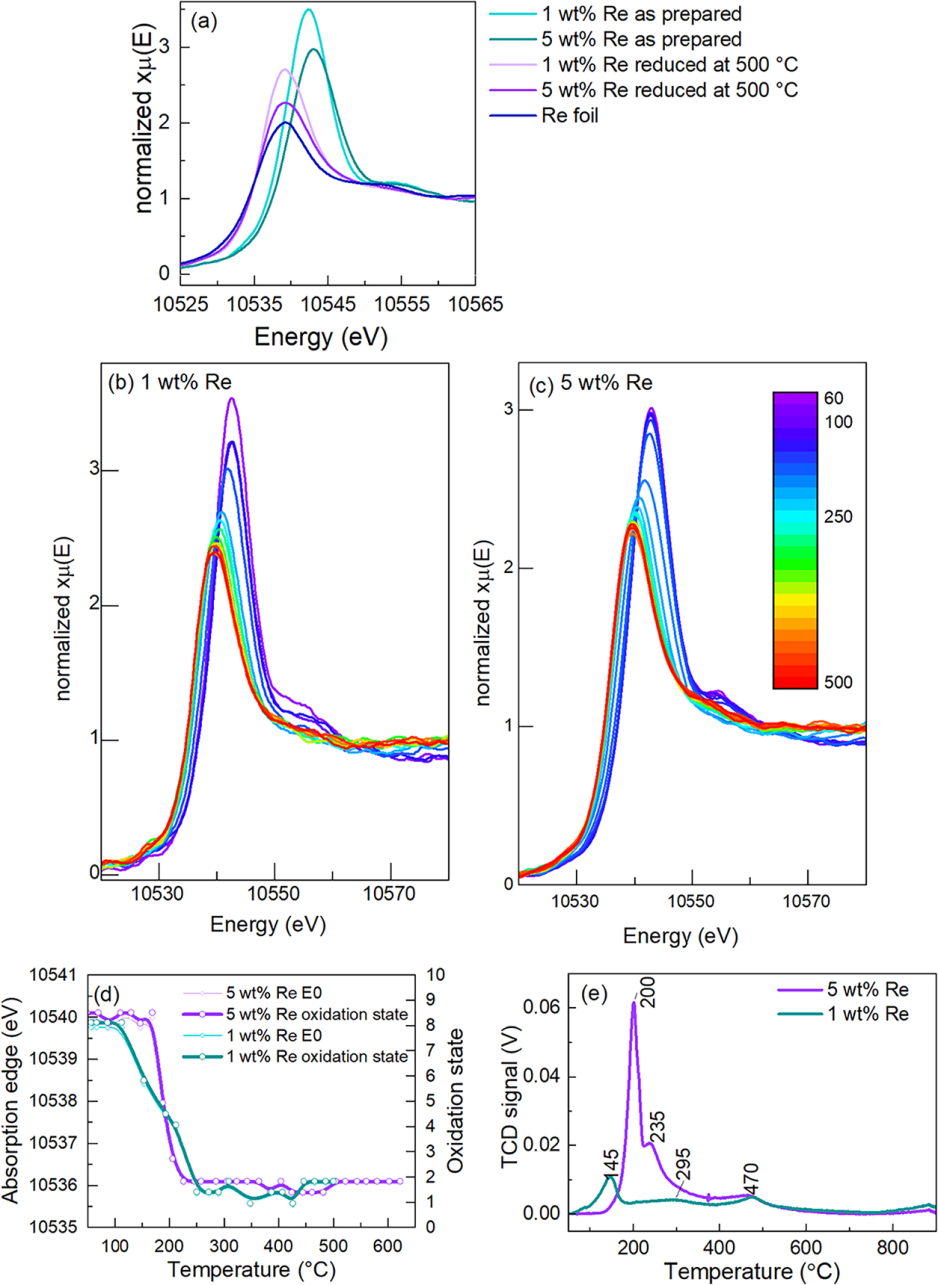
(a) Re L_3_-edge XANES of Re/TiO_2_ samples obtained at room temperature, (b, c) Re L_3_-edge XANES of 1 and 5 wt % Re/TiO_2_ catalysts measured during *in situ* H_2_-TPR, (d) absorption edge energy and oxidation state of rhenium in Re/TiO_2_ samples during reduction with H_2_, and (e) TCD signal of H_2_ consumption.

To relate the absorption edge position to the average Re oxidation state, the XAS of Re_2_O_7_, ReO_3_, ReO_2_, and metallic Re were obtained (Figure S6).^68^ The data suggest that both as-prepared 1 and 5 wt % Re/TiO_2_ catalysts have an average oxidation state of rhenium close to +7, and close to zero after *in situ* reduction at 500 °C. A linear regression of the *E*_0_ values of the rhenium standards (Figure S7) reveals that both 1 and 5 wt % Re/TiO_2_ catalysts have an average oxidation state of +1.2 after 1 h reduction at 500 °C under H_2_, cooling under He, and measured *in situ* at room temperature. Due to its large size and oxophilicity, rhenium only forms stable oxides of higher oxidation state, with ReO_2_ (Re^4+^) as the oxide of lowest rhenium oxidation state. ,,ReO“ or ,,Re_2_O“ only exists on the surface of metallic rhenium.^65,69^ Therefore, the XANES of the reduced Re/TiO_2_ catalysts suggest that their active sites are composed of metallic rhenium nanoclusters in close interaction with oxygen atoms from the TiO_2_ support.

XANES of Re L_3_-edge during temperature-programmed-reduction in 5% H_2_/He (H_2_-TPR) of the Re/TiO_2_ catalysts were performed to understand any differences in the reduction behavior of the two catalysts (Figure 3b,c). The main reduction event occurs in the 150–250 °C range for both catalysts, with the reduction of the 1 wt % Re starting at a slightly lower temperature, and over a larger temperature range. These results are in good agreement with the laboratory H_2_-TPR with H_2_ consumption measured by TCD (Figure 3e). The higher dispersion of rhenium clusters on 1 wt % Re/TiO_2_ can explain the lower reduction temperature at 145 °C, as more Re atoms are exposed to H_2_ in a smaller particle. The XPS analyses (Figure 1b) revealed that 1 wt % Re/TiO_2_ also contains rhenium species with a stronger interaction with the TiO_2_ support. These would be more resistant to reduction, which could explain the broader TPR peaks and the slower decrease in oxidation state with temperature.

In order to gather information about the rhenium species present during the catalysis, Re L_3_-edge XANES was also obtained *in operando* for the hydrogenation of CO_2_ over the 5 wt % Re/TiO_2_ catalyst, prereduced at 500 °C at 20 bar. The experimental conditions at the beamline were slightly different than those of the catalytic reaction evaluations, as the reaction gases were at lower pressure and diluted in helium. In all cases, there are only minor differences in the Re L_3_-edge XANES between those of the reduced catalyst and the operando spectrum (Figure S8).

The Re L_3_-edge EXAFS data of the catalysts reduced *in situ* at 500 °C, plotted as the magnitude of the Fourier transform (Figure 4), reveal that 5 wt % Re/TiO_2_ has a more significant contribution of metallic rhenium species than 1 wt % Re/TiO_2_, as indicated by the presence of a peak at 2.3 Å. This finding suggests that the catalyst with a higher percentage of rhenium contains more reduced rhenium species, despite the similarity in absorption edge energy, which is compatible with small metallic clusters interacting with oxygen atoms from TiO_2_. This suggests that 1 and 5 wt % Re/TiO_2_ have similar rhenium average oxidation states but exhibit an increase in cluster size with increasing rhenium amount, as reported for similar samples.^70,71^ The best fit EXAFS data for the 5 wt % Re/TiO_2_ is provided in Table 2 and Figure 5. (The respective *k*-space plots are given in Figure S9). The first Re–Re scattering path coordination numbers obtained for the 5 wt % catalysts prereduced at 250 or 500 °C were 2.3 ± 0.5 and 5.4 ± 1.1, respectively. The larger coordination number found for the sample reduced at a higher temperature indicates a larger Re cluster, which could also explain the lower white line intensity observed in the Re L_3_-edge XANES of Re/TiO_2_ reduced at the higher temperature, even though these samples presented the same absorption edge energy (Figure S10).^71^ Both samples presented a slight increase in average Re–Re coordination number after 5 h of reaction (200 °C and 20 bar), where a CN of 2.7 ± 0.7 (from 2.3 ± 0.5) was found for postreaction Re/TiO_2_ prereduced at 250 °C and 6.8 ± 1.1 (from 5.4 ± 1.1) for the sample prereduced at 500 °C. Unfortunately, the XAS data quality of the 1 wt % Re/TiO_2_ sample did not allow for a reliable EXAFS modeling due to the poor signal quality. Comparatively, Toyao et al.^26^ found a Re–Re CN of 3.4 for a 5 wt % Re/TiO_2_ catalyst prereduced also at 500 °C, but for a shorter time of 0.5 h as opposed to the 1-h reduction time employed in this study, and with a previous calcination step at 500 °C for 3 h in air. Yang et al.^70^ found an even lower Re–Re coordination number of 1.8 ± 0.4 for a similar 5 wt % Re/TiO_2_ sample prereduced at the same temperature of 500 °C for 0.5 h, but synthesized through the strong electrostatic adsorption impregnation method, which points to a significant effect of small changes in synthesis methodology on the nanostructure of the catalytic active site.

**Figure 4:**
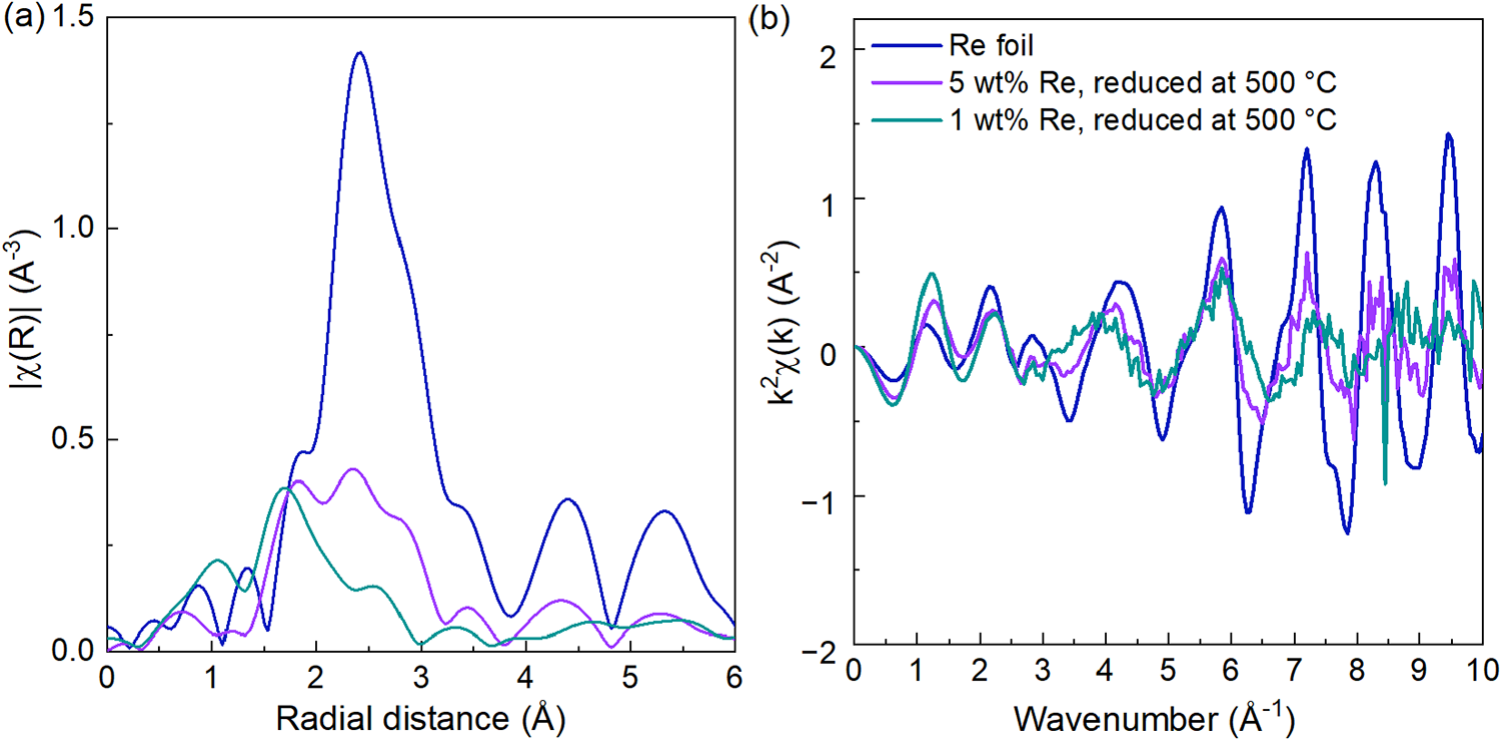
(a) Magnitude of the FT (*k* range 3–10.5 Å^–1^) and (b) *k*_2_-weighted Re L_3_-edge EXAFS in *k* space of Re/TiO_2_ of 5 or 1 wt % rhenium with *in situ* reduction under H_2_, compared to metallic Re.

**Figure 5:**
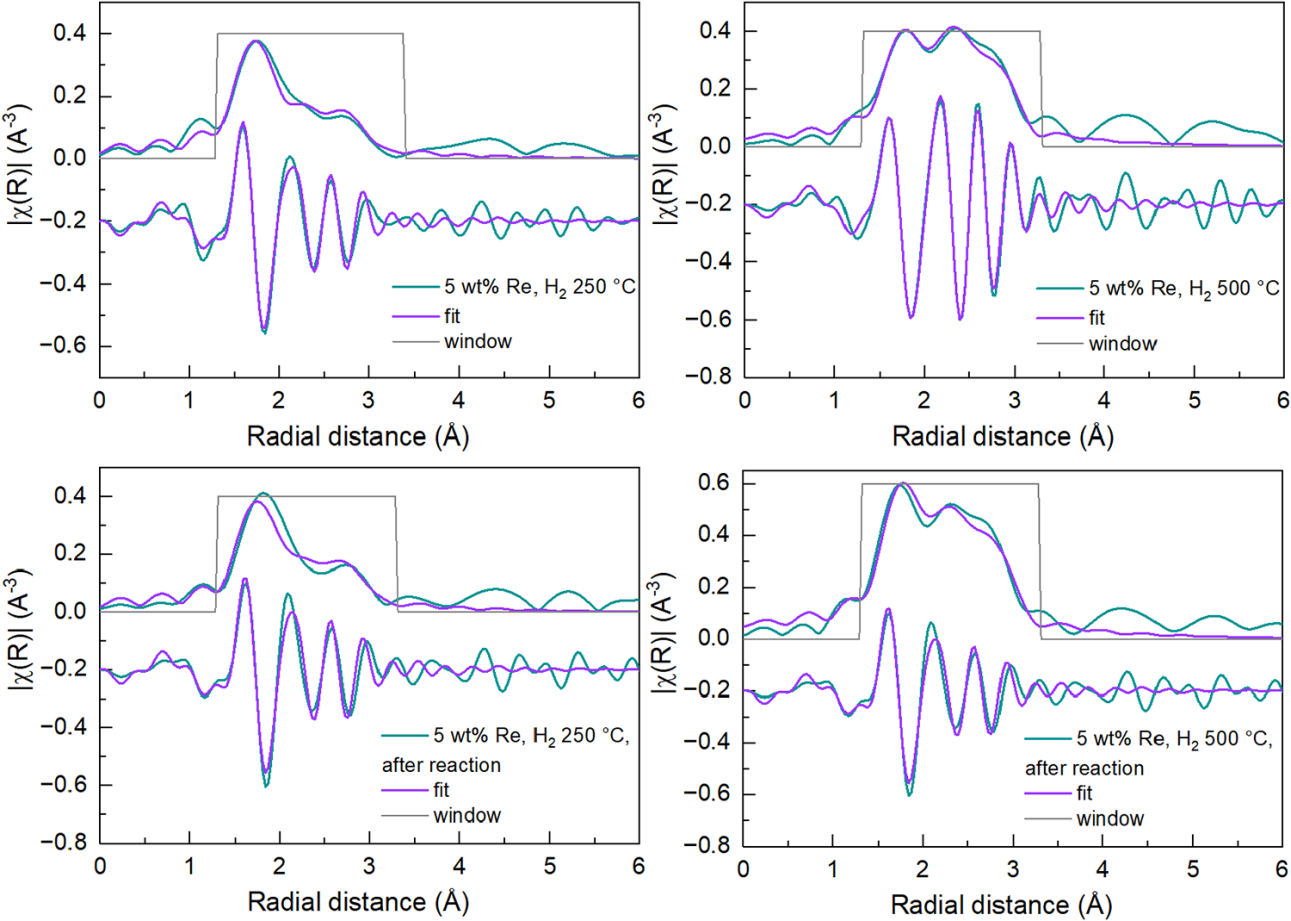
Best fit EXAFS models of Re/TiO_2_ samples. Magnitude (upper) and imaginary (lower) of the FT plotted in each panel. All data recorded at room temperature. Top left 5 wt % Re after 250 °C reduction. Top right 5 wt % Re after 500 °C reduction. Bottom left 5 wt % Re reduced at 250 °C after reaction. Bottom right 5 wt % Re reduced at 500 °C after reaction. Fit range 1.2 to 3.4 Å. The EXAFS fit in *k*-space is shown in SI.

**Table 2:** EXAFS Fitted Parameters for 5 wt % Re/TiO_2_ (*k* = 3–10.5 Å^–1^; *k* Weight = 1, 2, 3)

5 wt % Re/TiO_2_	path	CN	*R* (Å)	σ (10^–3^ Å^2^)	*E*_0_ (eV)
as prepared	Re–O	4.6 ± 0.7	1.71 ± 0.01	0.5 ± 1.3	2.4 ± 2.7
	Re–O	1.0 ± 0.6	2.10 ± 0.03	0.5 ± 1.3	2.4 ± 2.7
reduced at 250 °C	Re–Re	2.3 ± 0.5	2.73 ± 0.02	6.6 ± 1.3	5 ± 2
	Re–O	1.1 ± 0.2	2.08 ± 0.02	1.4 ± 2.3	14 ± 2
reduced at 500 °C	Re–Re	5.4 ± 1.1	2.76 ± 0.01	6.6 ± 1.3	5 ± 2
	Re–O	1.4 ± 0.4	2.11 ± 0.03	5.8 ± 2.8	14 ± 2
reduced at 250 °C–after reaction	Re–Re	2.7 ± 0.7	2.73 ± 0.02	6.6 ± 1.3	5 ± 2
	Re–O	1.2 ± 0.3	2.11 ± 0.02	1.4 ± 2.3	14 ± 2
reduced at 500 °C–after reaction	Re–Re	6.8 ± 1.1	2.76 ± 0.01	6.6 ± 1.3	5 ± 2
	Re–O	2.3 ± 0.5	2.12 ± 0.02	5.8 ± 2.8	14 ± 2

The DRIFTS analyses of the 1 and 5 wt % Re/TiO_2_ catalysts (Figure 6) in a flow of CO_2_/H_2_ (1:4 ratio) indicate the species adsorbed on the catalytic surface under a flow of CO_2_ and H_2_ diluted in argon at atmospheric pressure. Both samples present vibrational bands around 1350 and 1550 cm^–1^, which are almost entirely absent at 300 °C. These bands can be assigned as bidentate formate on TiO_2_.^31^ Both samples also present bands around 2000–1800 cm^–1^ that can be attributed to adsorbed CO species.^31,70,72^ These adsorbed CO bands appear at 150 °C and increase in intensity with temperature up to 250 °C and begin to decrease at 300 °C, which indicates that these species were formed under reaction conditions.

**Figure 6:**
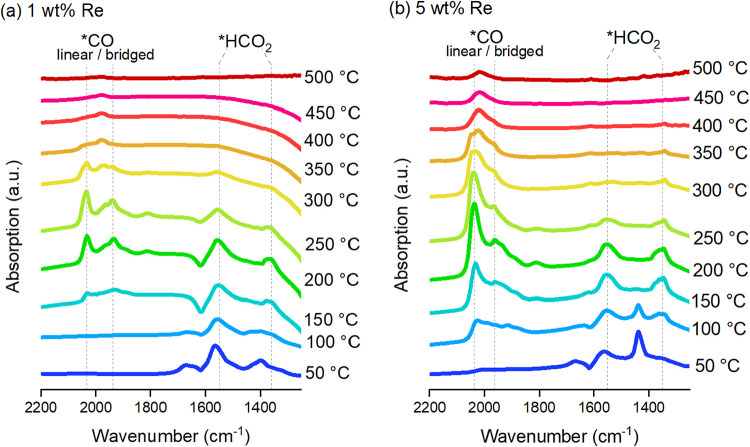
Diffuse reflectance infrared Fourier-transformed spectroscopy (DRIFTS) of Re/TiO_2_ with (a) 1 wt % of Re and (b) 5 wt % of Re prereduced under H_2_ at 500 °C and exposed to a mixture of CO_2_ and H_2_ (ratio of 1:4) at ambient pressure and various temperatures.

The sample with 5 wt % Re has a higher intensity of linearly adsorbed CO on the edge sites at 2038 cm^–1^ and lower intensity bands at 1963, 1881, and 1808 cm^–1^, which can be ascribed to bridged-CO adsorbed on perimeter Re–Ti sites or on two Re atoms.^70^ The 1 wt % sample has bands of similar intensity for linear CO at 2034 cm^–1^ and bands at 1968, 1938, and 1810 cm^–1^. The higher intensity of linearly adsorbed CO on the 5 wt % Re sample is expected due to the higher metal content, and thus higher particle size, whereas the 1 wt % Re sample may have more surface defects, which would facilitate the bridged configuration of adsorbed CO species.^70,73,74^ The IR bands between 2000–1800 cm^–1^ include vibrations from rhenium hydrides,^75,76^ rhenium carbonyls and bidentate adsorbed CO,^31^ which are difficult to distinguish due to their superpositions.

Table 3 presents data on the catalytic activity in the high-pressure hydrogenation of CO_2_ over Re/TiO_2_ with varying reduction temperatures and rhenium amounts. Generally, an increase in the reaction temperatures from 200 to 250 °C was detrimental to CH_3_OH selectivity, as more CH_4_ is formed at higher temperatures in this range, due to the kinetic limitations of the methanation reaction.^6^ At 150 °C, the selectivity is high, but the conversion of CO_2_ is low, even at 100 bar, as we have previously reported.^28^ Performing the reduction of the Re/TiO_2_ catalyst with 5 wt % Re at the higher temperature of 500 °C instead of 250 °C improves methanol productivity at both reaction temperatures of 200 and 250 °C. However, CH_4_ is still a problematic side product when the reaction is performed at 250 °C.

**Table 3:** Effect of Rhenium wt % and Temperatures of Pre-Reduction and Reaction on the High-Pressure Hydrogenation of CO_2_ over Re/TiO_2_ in a Fixed-Bed Reactor Packed 290 mg of Catalyst, *P =* 100 bar, CO_2_/H_2_ = 1:4, GHSV = 10,000 mL·g_cat_^–1^·h^–1^, Time on Stream = 6 h

				selectivity (%)			
Re wt %	*T* prereduction (°C)	*T* reaction (°C)	CH_3_OH STY^a^	CH_3_OH	CO	CH_4_	CO_2_ conversion (%)
1	250	200	15	80	<1	20	7
1	250	250	28	46	<1	53	21
1	500	200	55	99	<1	<1	19
1	500	250	65	97	1	1	23
5	250	150	2	97	<1	3	4
5	250	200	8	98	<1	2	18
5	250	250	13	65	<1	35	40
5	500	250	16	74	1	25	40
5	500	200	18	97	<1	3	33

a Space time yield (STY) calculated as grams of CH_3_OH produced per gram of Re per hour.

Meanwhile, the decrease in amount of rhenium from 5 to 1 wt % had a positive effect on methanol selectivity at both reaction temperatures, although the conversion of CO_2_ decreased. For 1 wt % Re/TiO_2_ reduced at 500 °C, a selectivity of 99% methanol at 19% CO_2_ conversion was achieved at 200 °C. At the same reaction conditions for 5 wt % Re/TiO_2_, a selectivity of 97% methanol at a CO_2_ conversion of 33% was obtained. Although conversion was higher with the catalyst with 5 wt % Re, the methanol space-time yield (STY) per gram of rhenium was higher for the catalyst with only 1 wt % (55 against 18 g_CH_3_OH_·g_Re_^–1^·h^–1^).

### Influence of Reaction Conditions for the High-Pressure Hydrogenation of CO_2_ to Methanol

The reaction conditions, including space velocities, pressures, and CO_2_/H_2_ ratios, were evaluated for their impact on CO2 conversion and methanol selectivity. The hydrogenation of CO_2_ was performed at 200 °C in a stainless steel fixed-bed flow reactor packed with Re/TiO_2_ catalysts reduced at 500 °C (Figure 7 and Table 4). The CO_2_ conversion was affected by all of the evaluated variables. A higher gas hourly space velocity (GHSV) of 40,000 mL·g_cat_^–1^·h^–1^ led to a conversion of 26% while a lower one of 10,000 mL·g_cat_^–1^·h^–1^ gave a conversion of 33%, which is expected since higher space velocities give shorter contact times on the catalyst. Higher pressures significantly increased CO_2_ conversion, which was 5% at 20 bar and 33% at 100 bar. Thermodynamically, increasing pressures positively affect methanol formation from CO_2_.^28^

**Figure 7:**
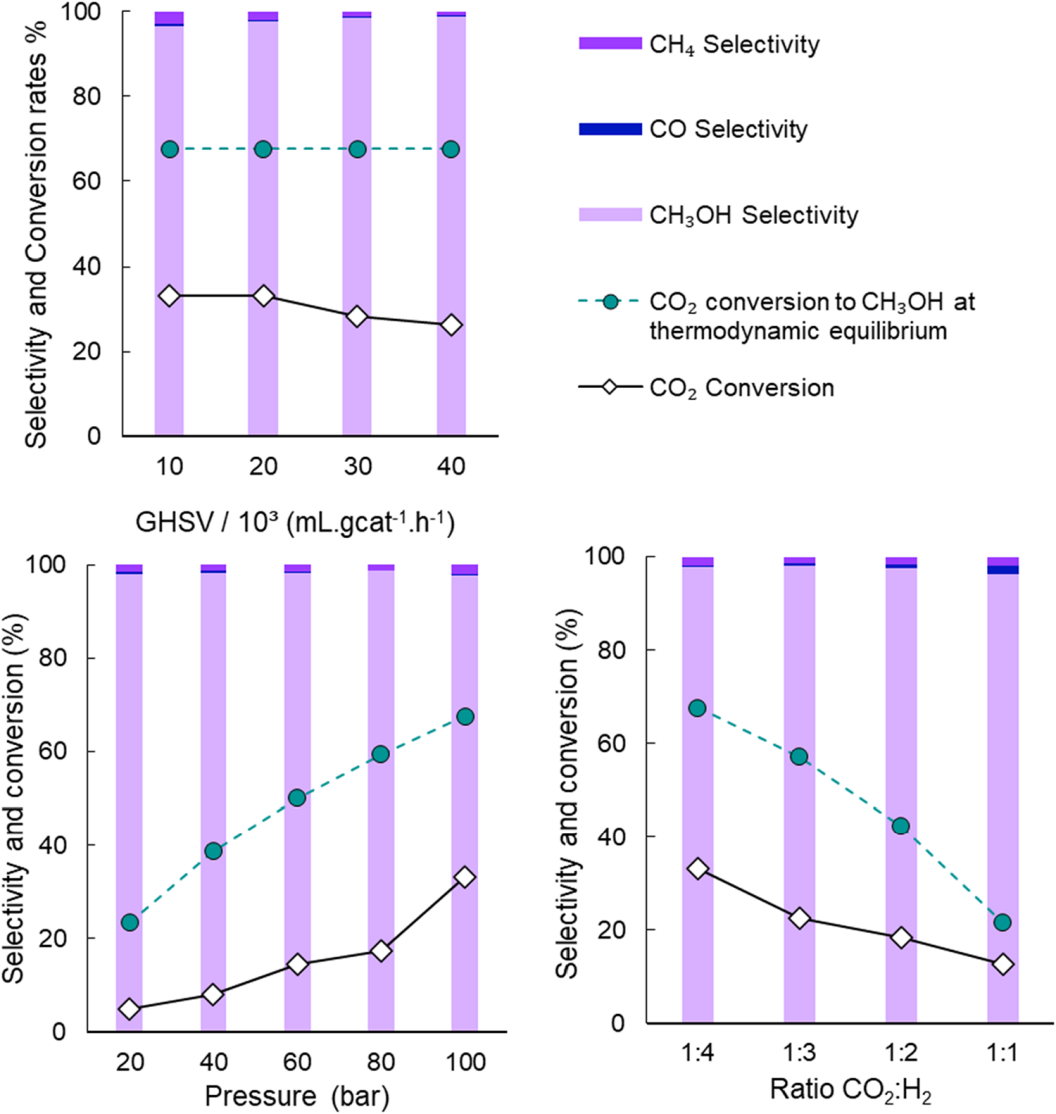
High pressure hydrogenation of CO_2_ over 5 wt % Re/TiO_2_. Reaction conditions (unless otherwise specified): fixed-bed reactor packed 290 mg of 5 wt % Re/TiO_2_ prereduced at 500 °C, *P* = 100 bar, CO_2_/H_2_ = 1:4, *T =* 200 °C; GHSV = 20,000 mL·g_cat_^–1^·h^–1^. Thermodynamic equilibrium conversion considered only CO_2_ to CH_3_OH.

**Table 4:** Catalytic Performance of Re/TiO_2_ on the High-Pressure Hydrogenation of CO_2_ to CH_3_OH^a^ ^b^,,^c^

						selectivity				
Re (wt %)	*T* prereduction (°C)	*T* reaction (°C)	pressure (bar)	CO_2_/H_2_	CH_3_OH STY^c^	CH_3_OH	CO	CH_4_	conversion of CO_2_	GHSV^c^ (10^3^)
1	250	200	100	1:4	15	80	<1	20	7	10
1	250	250	100	1:4	28	46	<1	53	21	10
1	500	200	100	1:4	55	99	<1	<1	19	10
1	500	250	100	1:4	65	97	1	1	23	10
5	250	150	100	1:4	2	97	<1	3	4	10
5	250	200	100	1:4	8	98	<1	2	18	10
5	250	250	100	1:4	13	65	<1	35	40	10
5	250	250	100	1:4	30	89	1	11	29	20
5	250	250	100	1:4	49	94	1	5	22	40
5	500	250	100	1:4	16	74	1	25	40	10
5	500	200	100	1:4	18	97	<1	3	33	10
5	500	200	100	1:4	37	98	<1	2	33	20
5	500	200	100	1:4	48	99	<1	1	28	30
5	500	200	100	1:4	59	99	<1	1	26	40
5	500	200	80	1:4	20	99	<1	1	17	20
5	500	200	60	1:4	16	98	<1	1	14	20
5	500	200	40	1:4	9	98	1	1	8	20
5	500	200	20	1:4	6	98	1	1	5	20
5	500	200	100	1:3	32	98	<1	1	22	20
5	500	200	100	1:2	35	98	1	2	18	20
5	500	200	100	1:1	36	96	2	2	13	20

a Reaction conditions: fixed bed reactor packed with 290 mg of Re/TiO_2_.

b Space time yield (STY) calculated as grams of CH_3_OH produced per gram of Re per hour.

c Gas hourly space velocity (GHSV) calculated as mL·g_cat_^–1^·h^–1^

Ratios of CO_2_/H_2_ with excess hydrogen, such as 1:4, gave high CO_2_ conversion. While the catalytic performances at higher space velocities and CO_2_-rich reactant mixtures show slightly lower conversions, these are still interesting from an industrial perspective, as they allow for a greater amount of CO_2_ to be processed at a given time. At pressures between 20 and 100 bar at *T =* 200 °C, none of the aforementioned variables had a significant impact on reaction selectivity, attesting to the suitability of Re/TiO_2_ as an effective catalyst for CO_2_ hydrogenation to CH_3_OH. Remarkably, the methanol selectivity was still high (96%) even at unfavorable conditions such as CO_2_/H_2_ = 1:1 (Figure 7). Methanol selectivity was also stable at 98–99% through the range of higher pressures of 20–100 bar, and only varies from 97 to 99% when increasing GHSV from 10,000 to 40,000 mL·g_cat_^–1^·h^–1^. Yet, it is noteworthy that at higher temperatures or ambient pressure, selectivity shifts to CH_4_ or CO, respectively, as shown by previous work.^28^

The thermodynamic equilibrium was evaluated through minimization of Gibbs free energy, where H_2_, CO_2_, CO, CH_3_OH, CH_4_, and H_2_O were considered as components. The CO_2_ conversion and selectivities obtained with Aspen Plus showed that, at equilibrium, methane selectivity is >99.9% in all scenarios evaluated, at a pressure range of 1–120 bar, temperature from 150 to 300 °C and CO_2_/H_2_ ratio of 1:1 through 1:4 and any combination of these variables (Figures S11–S16). This was expected, since methane is the most thermodynamically stable product.^77–81^ Based on Le Châtelier’s principle, reactions where the number of moles is reduced, such as the production of methanol and methane from the hydrogenation of carbon dioxide, are favored by high pressures.^82–84^ Additionally, lower temperatures benefit methane formation as can be seen in Figure 8(a),(b). On the other hand, between 150 and 300 °C, higher temperatures improve the selectivity at thermodynamic equilibrium for methanol and carbon monoxide, as shown in Figure 8(b),(d). Moreover, while increased pressure favors methanol selectivity, it negatively impacts that of carbon monoxide. Although these conditions enhance CO_2_ conversion, they also promote methane formation. High pressure and low temperature hinder carbon deposition and favor methanation.^85,86^ Despite thermodynamic equilibrium predictions favoring CH_4_ formation, the Re/TiO_2_ catalyst achieved notable methanol selectivity, particularly at higher GHSV, pressures, and high CO_2_ to H_2_ ratios such as 1:4 (Figure 7). This difference suggests that the catalyst has a significant influence on directing the reaction selectivity toward CH_3_OH, probably because of kinetic constraints in the methanation pathway and the unique active sites offered by Re/TiO_2_.

**Figure 8:**
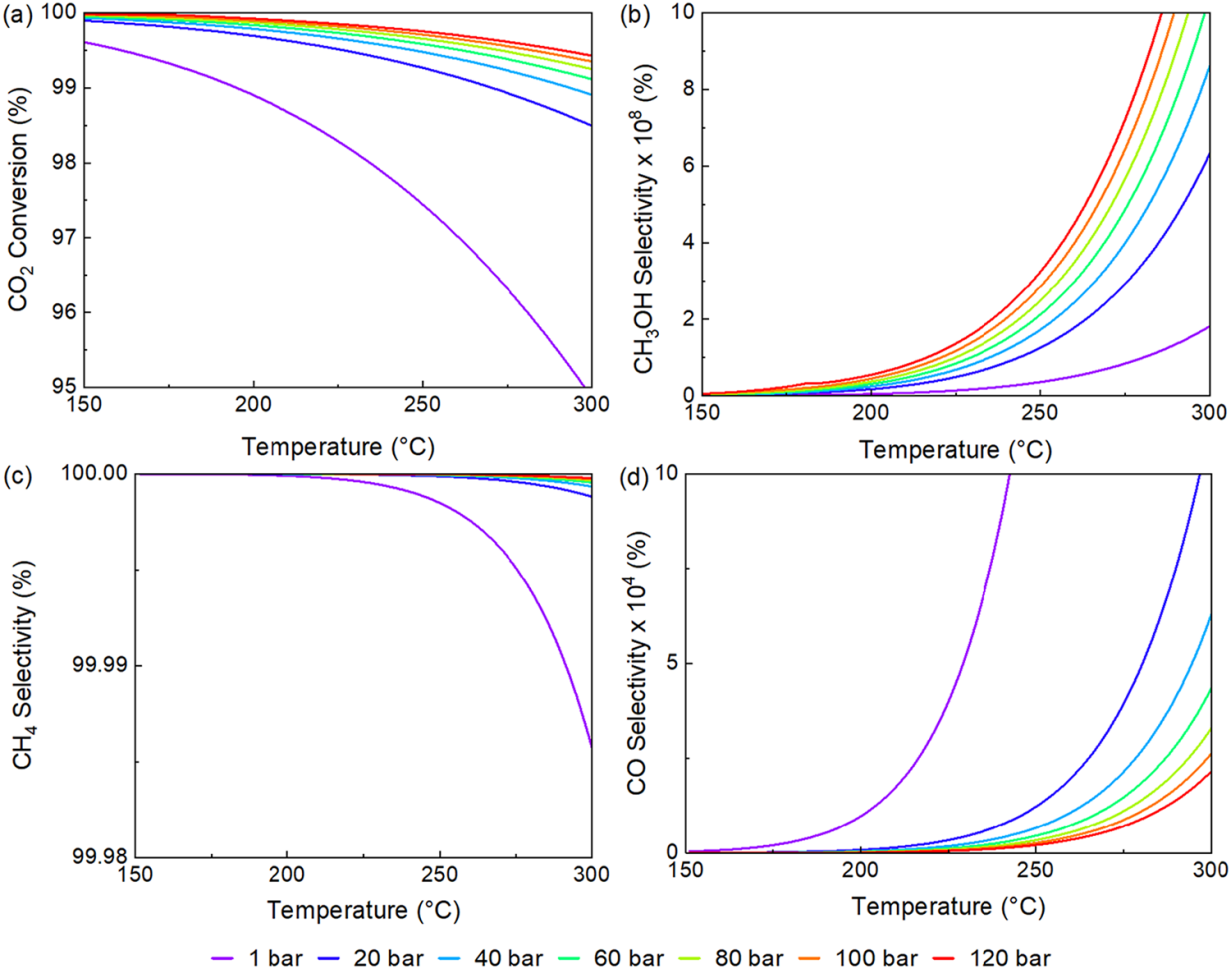
Thermodynamic equilibrium simulated (a) CO_2_ conversion, (b) CH_3_OH selectivity, (c) CH_4_ selectivity and (d) CO selectivity.

Additionally, Figure 7 shows the CO_2_ conversion curves for experimental data and thermodynamic equilibrium considering only methanol as the reaction product. It is noted that the GHSV does not influence the thermodynamic equilibrium since the minimization of Gibbs free energy method does not use information about the interactions of the reactants/products with the active sites of the catalyst used. In all cases, the theoretical conversion is higher than the experimental one; however, these curves are similar in their behavior, especially for the pressure and ratio (CO_2_/H_2_) variables. More detailed information about the thermodynamic equilibrium can be found in the Supporting Information.

### Theoretical Investigations

To better understand the structure-performance relationship of our TiO_2_-supported Re catalysts for CO_2_ hydrogenation to methanol, DFT calculations were performed. Five theoretical models were constructed, and two steps of the mechanism were evaluated as previously described by Shen and co-workers for a similar catalyst.^32^ The models included a single atom **Re**_**1**_/TiO_2_(101), a four atoms cluster **Re**_**4**_/TiO_2_(101), a ten atoms cluster **Re**_**10**_/TiO_2_(101), a Re monolayer (**Re**_**layer**_) covering the TiO_2_(101) surface, and the pristine **TiO**_**2**_(101) surface (Figure 9a for top view and Figure S17 for side view). In all cases, when necessary, the most stable adsorption sites for the rhenium atom, the clusters, or the adsorbates were determined. The optimized geometry revealed Re–O bond lengths ranging from 1.95 to 2.11 Å, with shorter distances associated with **Re**_**1**_/TiO_2_(101) and longer distances linked to **Re**_**10**_/TiO_2_(101). These values are similar to those obtained from the best-fit EXAFS models of the reduced Re/TiO_2_ samples, which had R values of 2.08 and 2.11 Å for the Re–O bonds when reduced at 250 and 500 °C, respectively. This further indicates the structure of Re/TiO_2_ resembles metallic Re nanoclusters in close interaction with the TiO_2_ support through O atoms. Figure S18 shows the electron density difference for all Recontending models. Each case displays distinct chemical environments for electron transfer, indicating variations in the active sites of these catalysts for the reaction.

**Figure 9:**
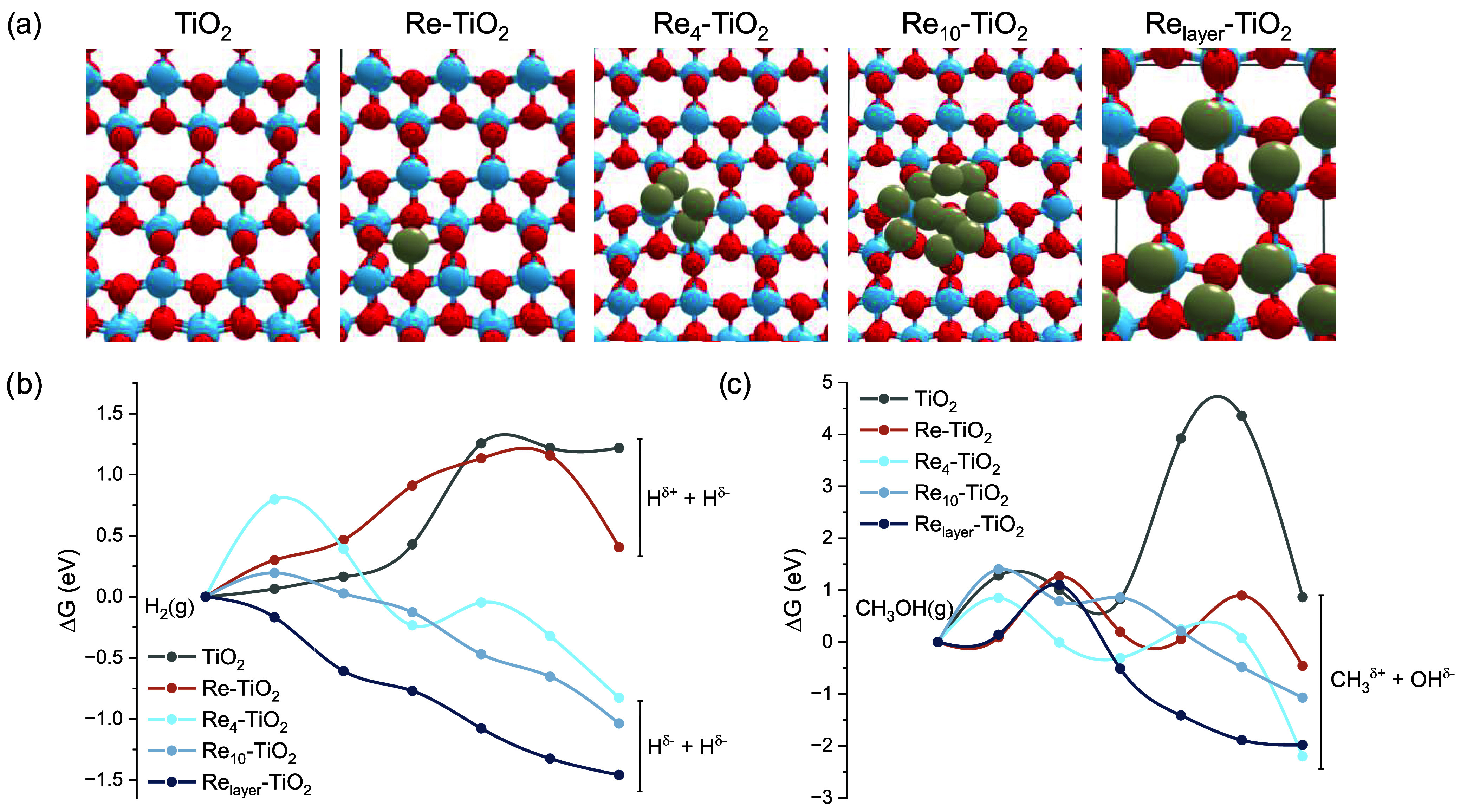
Theoretical models (a) and free energy profiles for the reaction routes of H_2_ (b) and CH_3_OH (c) dissociation on different model catalysts.

The first evaluated step of the mechanism of CO_2_ conversion to methanol was the dissociation of the hydrogen molecule, which is fundamental to generating adsorbed hydrogen atoms over the surface involved in the reduction steps. The H_2_ molecule adsorption starts this step, so the Gibbs free energy of adsorption (Δ*G*_ads_) was determined for each model. As shown in Table 1, the H_2_ molecule adsorption energy increases from **Re**_**1**_ to **Re**_**10**_ and then shows intermediate values to **Re**_**layer**_, as well as to pristine **TiO**_**2**_. Such variation of the Δ*G*_ads_ can be correlated with the coordination number of the rhenium atom. **Re**_**1**_ with the smaller coordination number exhibits a larger Δ*G*_ads_, which can be associated with chemical adsorption, corroborated by the distinct adsorption mode observed (side-on, as shown in Figure S19). This process seems to be like an oxidative addiction to the rhenium atom. For **Re**_**10**_ and **Re**_**layer**_, each rhenium atom displays a higher coordination number, and lower values of Δ*G*_ads_ are observed.

The Gibbs free energy (Δ*G*) for the H–H dissociation and the activation energy for this reaction were also calculated for each model (see Figures S20 and S21 for product configurations and transition states (TS), respectively). In this case, the importance of rhenium inclusion is demonstrated in the reaction free energy, which is lower in all rhenium-included models than in pristine **TiO**_**2**_. For this reaction, as the coordination number of rhenium increases, the reaction energy becomes more spontaneous, and the reaction is endergonic only for **Re**_**1**_. The reason for such a decrease in reaction energy can be suggested by analyzing the charge density differences (Figure S22). For **Re**_**1**_ and **TiO**_**2**_, the charge density indicates the formation of H^δ+^ and H^δ‑^ species, and therefore, the reaction goes through a heterolytic pathway. In both cases, the H^δ+^ in the products is bonded to an oxygen atom of the surface, and the TS is observed when the hydrogen approaches this atom, requiring a weakening of the Ti–O bond and displaying larger values of *E*_a_ (Table 5). On the other hand, the charge densities over the clusters indicate the formation of an H^δ−^–H^δ−^ pair and a homolytic pathway. For these last three models, the TS is observed at the beginning of the reaction coordinate when the dissociation of the H_2_ bond starts. For **Re**_**4**_ and **Re**_**10**_, the *E*_a_ shows the same trend as the Δ*G*_ads_, indicating a correlation between the coordination number, the amount of charge transferred between the species, and the energy required to break such an interaction partially. For **Re**_**layer**_, the entire dissociation pathway is exergonic.

**Table 5:** Adsorption Free Energy, Dissociation Free Energy, and Activation Barrier for the Models Studied in this Work

	Δ*G*_ads_ (eV)	Δ*G*_dissociation_ (eV)	*E*_a_ (eV)
H_2_			
**Re**_**1**_	–1.55	0.41	1.16
**Re**_**4**_	–0.61	–0.83	0.80
**Re**_**10**_	0.64	–1.04	0.20
**Re**_**layer**_	0.47	–1.53	0.00
**TiO**_**2**_	0.61	1.05	1.26
CH_3_OH			
**Re**_**1**_	0.17	–0.46	1.27
**Re**_**4**_	–0.24	–2.20	0.85
**Re**_**10**_	1.31	–1.07	1.40
**Re**_**layer**_	1.08	–2.42	1.10
**TiO**_**2**_	0.16	0.86	4.36

Subsequently, the H_3_C–OH dissociation step was evaluated. In this case, if methanol is formed in the reaction, the desorption energy can be compared to the activation energy of bond dissociation to determine whether the molecule is released or tends to form species like CH_3_^δ+^, which can react and lead to other products, such as CH_4_. The Δ*G*_ads_ of methanol over these models (Figure S23) were obtained and shown in Table 5. The C–O bond dissociation is also endergonic only over pristine **TiO**_**2**_, while for the other models, an exergonic reaction is observed (see Figure S24 and S25 for products and TS, respectively). Similarly to H_2_ dissociation over **Re**_**1**_ and **TiO**_**2**_, the positively charged species CH_3_^δ+^ in the products (Figure S26 for charge differences plot) is bonded to oxygen from the TiO_2_ surface, and a weakening of the Ti–O bond is required. The *E*_a_ energy increases from **Re**_**4**_ to **Re**_**10**_ and then decreases to **Re**_**layer**_. In both **Re**_**4**_ and **Re**_**layer**_, the dissociation reaction occurs at the top atoms of the cluster or the **Re**_**layer**_. In these cases, the pathway for the charged CH_3_^δ+^ species shows shorter distances to the rhenium atoms compared to **Re**_**10**_, where the path proceeds from the top to the side atoms of the cluster. The interaction of the charged CH_3_^δ+^ species with more rhenium atoms resulted in lower activation energy.

These calculations are in line with the selectivity observed experimentally. The **Re**_**layer**_ model is the best fit for 5 wt % rhenium, consistent with nanoparticles of greater than 1 nm on the TiO_2_. The **Re**_**layer**_ exhibits no barrier to H_2_ dissociation and more catalytic sites, in agreement with the higher overall conversion observed for the 5 wt % rhenium material. The largest CH_4_ formation, and consequent lowest selectivity to CH_3_OH, was observed on the catalyst with the lowest particle size, 1 wt % Re/TiO_2_ reduced at 250 °C, which could be related to the simulated **Re**_**4**_. The dissociation of methanol is spontaneous for all rhenium-containing species, but the most negative values are found for **Re**_**layer**_ and **Re**_**4**_. The desorption of CH_3_OH, however, is not spontaneous for **Re**_**4**_ (as Δ*G*_desorption_ = −Δ*G*_ads_), therefore leading to a low selectivity. In fact, the highest methanol selectivity is observed for the catalyst with the second smallest size of ∼0.6 nm subnanometric Re clusters, 1 wt % Re reduced at 500 °C. In this case, 1 wt % Re/TiO_2_ reduced at 500 °C agrees very well with **Re**_**10**_, where the desorption energy of CH_3_OH is spontaneous. At the same time, the *E*_a_ for C–O bond dissociation for **Re**_**10**_ is endergonic, thus indicating that the formed methanol tends to be released instead of forming charged species that can react and lead to other products, which would decrease the methanol yield. For the **Re**_**layer**_, the desorption of methanol occurs spontaneously, and the activation barrier is endergonic. In contrast, the dissociation of methanol in the **Re**_**layer**_ is highly exergonic, resulting in the stabilization of the CH_3_^δ+^ species. These stabilized species can then react to enhance the yield of CH_4_. This finding is consistent with the observed increase in CH_4_ yield when comparing the 5 wt % rhenium catalyst to the 1 wt % rhenium catalyst.

## Conclusion

The Re/TiO_2_ catalysts are effective for the hydrogenation of CO_2_ to methanol across a wide range of pressures and space velocities. The synthesis of Re/TiO_2_ with varied rhenium amounts generates different rhenium species in the as-prepared catalysts, as well as when they are reduced under H_2_. The lower rhenium loading of 1 wt % results in smaller clusters with stronger interactions with the support, which are subnanometric in size, whereas 5 wt % Re/TiO_2_ forms particles around 1 nm in diameter. Yet, both 1 and 5 wt % Re/TiO_2_ present very similar average oxidation states of approximately +1, which is compatible with small metallic Re clusters with interaction to oxygens of the TiO_2_ support, as shown by bond lengths calculated by DFT and measured by EXAFS. DFT calculations also revealed that the larger the Re cluster size, the lower the energy barriers for H_2_ activation, in line with the higher conversion rates observed for the larger Re particles. However, the correlation of size and CH_3_OH selectivity is not straightforward, as the energy of CH_3_OH dissociation and subsequent hydrogenation to CH_4_ is lower both over larger **Re**_**layer**_ nanoparticles and over a rather small cluster **Re**_**4**_. An optimal cluster size of **Re**_**10**_ leads to a more spontaneous CH_3_OH desorption and thus higher selectivity. Moreover, the thermodynamics of CO_2_ hydrogenation are highly selective to CH4, indicating that kinetic effects and catalysis are key factors in methanol synthesis from CO_2_. At 200 °C, where CH_4_ formation is kinetically hindered, 1 and 5 wt % Re/TiO_2_ catalysts were very selective (99 and 97% CH_3_OH). A slight increase in temperature to 250 °C highlights the effect of Re cluster size, with 5 wt % Re/TiO_2_ achieving 74% selectivity and 1 wt % Re/TiO_2_ achieving 97% selectivity to CH_3_OH. In sum, while a higher rhenium loading will increase the yield of a Re/TiO_2_ catalyst, adequate cluster sizes are vital for selective methanol production.

## Supplementary Material



## Notes

Additional spectra and TEM images for the Re/TiO_2_ catalysts; detailed methodology for the thermodynamic equilibrium calculations and additional plots; additional images of the theoretical models presented in the DFT study (PDF)
