# Supplementary material for: Operando XAS and DFT Uncover Structure-Performance Relationships in Re/TiO2 for Selective CO2 Hydrogenation to Methanol
Source: ACS Catal. 2025 Nov 4;15(22):19111–26. doi: 10.1021/acscatal.5c05984 (PMC12645472; doi:10.1021/acscatal.5c05984)
Supplement: Supplementary file 1 [file cs5c05984_si_001.pdf]

Supporting Information

Operando XAS and DFT Uncover Structure-Performance Relationships in Re/TiO_2 for Selective CO_2 Hydrogenation to Methanol

Maite Lippel Gothe[a]*, Adriano Henrique Braga[a], Lais Reis Borges[a], Jiyun Hong[b], Giliandro Farias[c], Alvaro David Torrez Baptista[c], Bryan Alberto Laura Larico[a], Ana Barbara Moulin Cansian[a], Caetano Rodrigues Miranda[c], Simon R Bare[b], Liane Marcia Rossi[a], Pedro Vidinha[a]*.

[a] ML Gothe, AH Braga, LR Borges, BAL Larico, ABM Cansian, LM Rossi, P Vidinha
Departamento de Química Fundamental, Instituto de Química
Universidade de São Paulo
Av Prof Lineu Prestes 748, São Paulo, SP, Brazil
E-mail: maite.gothe@usp.br and pvidinha@iq.usp.br

[b] J Hong, SR Bare
Stanford Synchrotron Radiation Lightsource
SLAC National Accelerator Laboratory
Menlo Park 94025, CA, United States of America

[c] G Farias, ADT Baptista, CR Miranda
Instituto de Física
Universidade de São Paulo
R. do Matão, 1371, São Paulo, SP, Brazil

List of figures

Figure S1. Raman spectra of rhenium standards and rhenium catalysts Re/TiO_2 with 5 and 1 wt% Re as prepared 3
Figure S2. Rietveld refinement from XRD of Re/TiO_2 catalysts containing (a) 1 wt% of Re and (b) 5 wt% of Re, pre-reduced at 500 °C under H_2. Rhenium peaks are marked ♦. 4
Figure S3. HR-STEM of 1 wt% Re/TiO_2 reduced at 250 °C under H_2........ 5
Figure S4. Particle size distribution of Re NPs on Re/TiO_2 evaluated through measurement of >200 particles seen by HR-STEM. 6
Figure S5. Normalized 1st derivative of Re L_3-edge XANES of Re/TiO_2 samples and Re foil standard. 6
Figure S6. Re L_3-edge XANES comparison of Re/TiO_2 to Re standards. 7
Figure S7. Oxidation state of Re as a function of absorption edge energy 7
Figure S8. 1st derivative of $\mu(E)$ obtained from operando XANES of CO_2 hydrogenation reaction over Re/TiO_2: (a) 1 wt% Re, pre-reduced at 500 °C; (b) 5 wt% Re, pre-reduced at 250 °C; (c) 5 wt% Re, pre-reduced at 500 °C. Reaction conditions: fixed-bed quartz reactor packed 70 mg of Re/TiO_2, P = 20 bar, CO_2:H_2 = 1:4, T = 200 °C, GHSV = 10,000 mL.g_{cat}^{-1}.h^{-1}........ 8
Figure S9. The k_2-weighted Re L_3-edge EXAFS in k space are shown for Re/TiO_2 with 5 wt% Re (top left) reduced at 250 °C, (top right) reduced at 500 °C, (bottom left) after reaction of 250°C reduced, (bottom right) after reaction of 500°C reduced. The data is shown in green, and the EXAFS best fit is shown in purple. The k-window of 3-11 Å^{-1} used for Fourier transform is also shown in black........ 8

Figure S10. Room-temperature measurements of Re L_3-edge XANES of Re/TiO_2 reduced at 250 °C and 500 °C plotted in (a) normalized μ(E) and (b) normalized 1st derivative of μ(E). 9
Figure S11. Thermodynamic equilibrium simulated for calculating CO_2 convertion (%) using ratio 1:4 (CO_2:H_2) (a) considering CO_2, H_2, CH_3OH, CO, and CH_4 (b) considering CO_2, H_2, CH_3OH, and CO. 13
Figure S12. Log_{10} of Selectivity (%) at thermodynamic equilibrium for CH_3OH, CH_4, and CO using ratio 1:1 (CO_2:H_2) at different pressures (a) 1 bar (b) 20 bar (c) 40 bar (d) 60 bar (e) 80 bar (f) 100 bar (g) 120 bar. 15
Figure S13. Log_{10} of Selectivity (%) at thermodynamic equilibrium for CH_3OH, CH_4, and CO using ratio 1:2 (CO_2:H_2) at different pressures (a) 1 bar (b) 20 bar (c) 40 bar (d) 60 bar (e) 80 bar (f) 100 bar (g) 120 bar. 16
Figure S14. Log_{10} of Selectivity (%) at thermodynamic equilibrium for CH_3OH, CH_4, and CO using ratio 1:3 (CO_2:H_2) at different pressures (a) 1 bar (b) 20 bar (c) 40 bar (d) 60 bar (e) 80 bar (f) 100 bar (g) 120 bar. 17
Figure S15. Log_{10} of Selectivity (%) at thermodynamic equilibrium for CH_3OH, CH_4, and CO using ratio 1:4 (CO2:H2) at different pressures (a) 1 bar (b) 20 bar (c) 40 bar (d) 60 bar (e) 80 bar (f) 100 bar (g) 120 bar. 18
Figure S16. Log_{10} of Selectivity (%) at thermodynamic equilibrium for CH_3OH, CH_4, and CO using ratio 1:5 (CO2:H2) at different pressures (a) 1 bar (b) 20 bar (c) 40 bar (d) 60 bar (e) 80 bar (f) 100 bar (g) 120 bar. 19
Figure S17. Side view of theoretical models studied in this work. 20
Figure S18. Top and side view charge density difference for the interaction between Re, Re_4, Re_{10}, and Re_{layer} with TiO_2. Green indicates negative charge density, and red indicates positive charge density. 20
Figure S19. Top and side view of H_2 molecule adsorption in the theoretical models studied in this work. 21
Figure S20. The top and side views of H_2 molecules after dissociation in the theoretical models studied in this work. 21
Figure S21. Top and side view of TS state for H_2 molecule dissociation in the theoretical models studied in this work. 22
Figure S22. Top and side view charge density difference for H_2 molecule after dissociation in the theoretical models studied in this work. Green indicates negative charge density, and red indicates positive charge density. 22
Figure S23. Top and side view of CH_3OH molecule adsorption in the theoretical models studied in this work. 23
Figure S24. The top and side views of the CH_3OH molecule after dissociation in the theoretical models studied in this work. 23
Figure S25. Top and side view of TS state for CH_3OH molecule dissociation in the theoretical models studied in this work. 24
Figure S26. Top and side view charge density difference for CH_3OH molecule after dissociation in the theoretical models studied in this work. Green indicates negative charge density, and red indicates positive charge density. 24

Catalyst characterizations

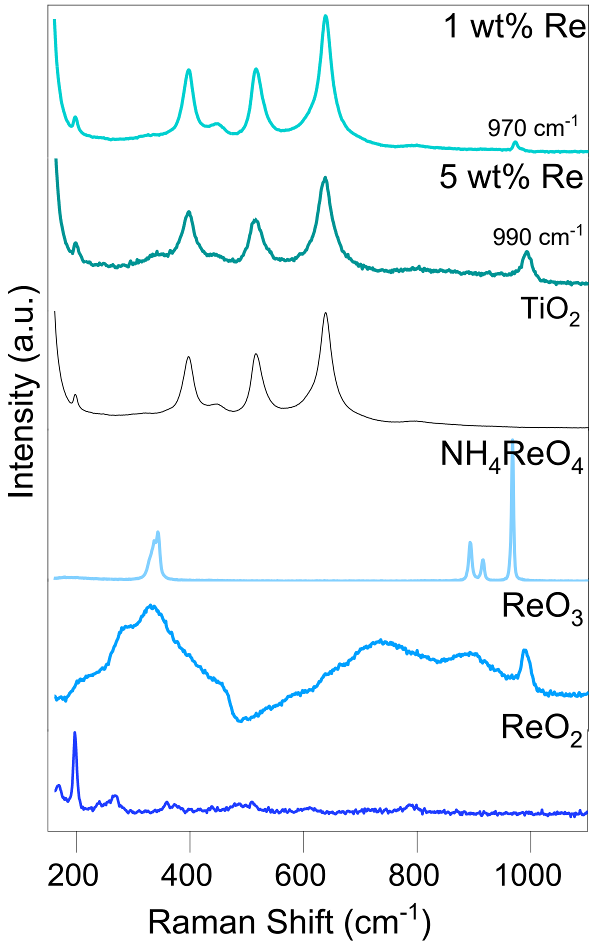

Figure S1. Raman spectra of rhenium standards and rhenium catalysts Re/TiO_2 with 5 and 1 wt% Re as prepared

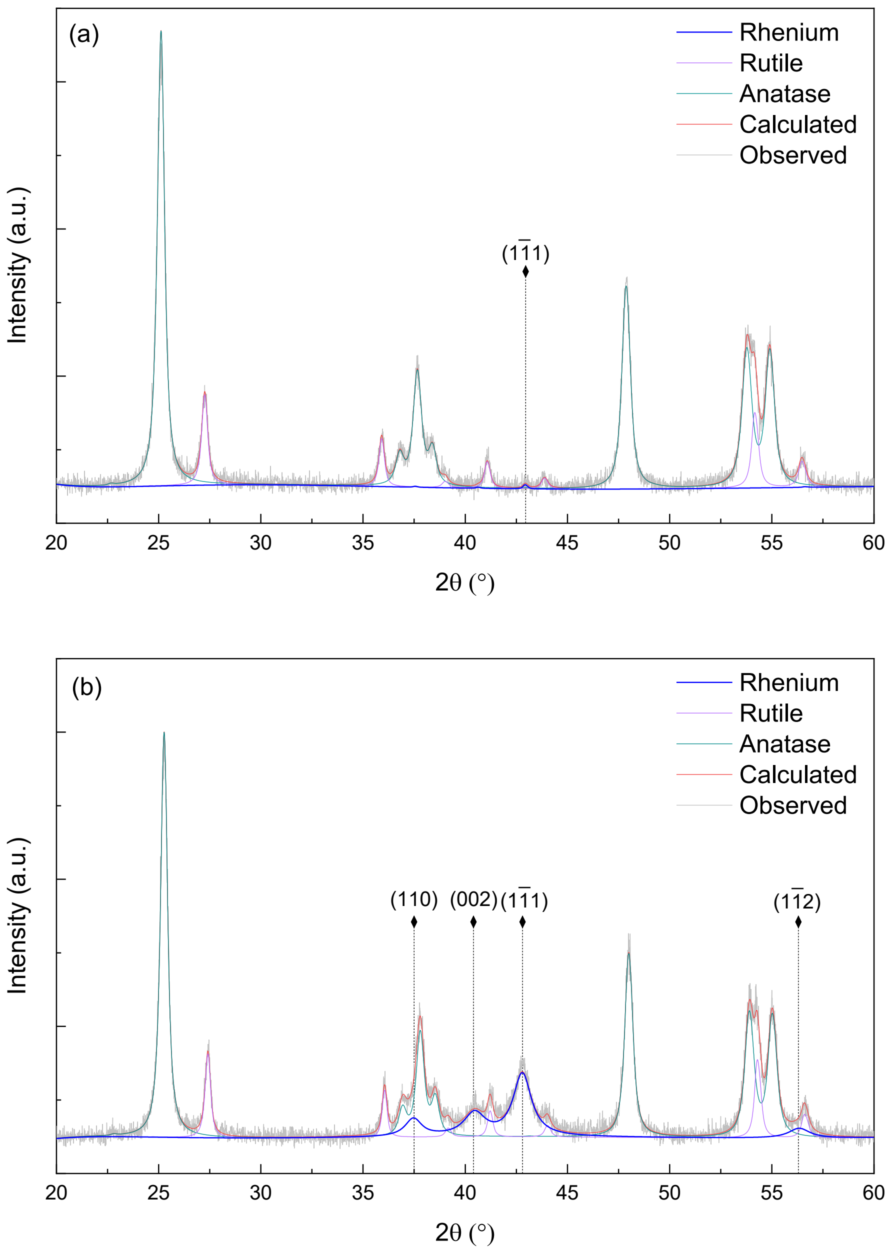

Figure S2. Rietveld refinement from XRD of Re/TiO_2 catalysts containing (a) 1 wt% of Re and (b) 5 wt% of Re, pre-reduced at 500 °C under H_2. Rhenium peaks are marked ♦.

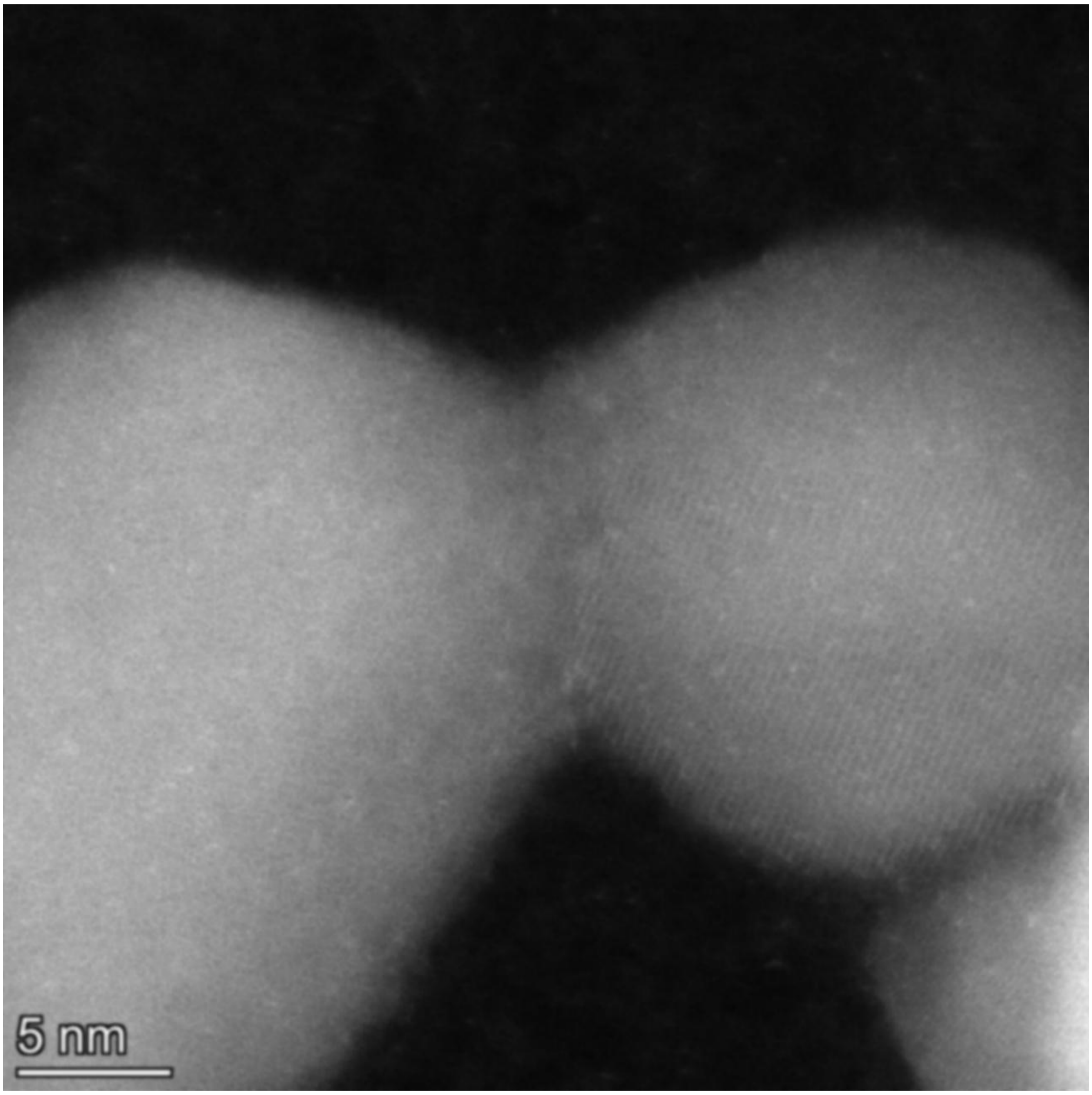

Figure S3. HR-STEM of 1 wt% Re/TiO_2 reduced at 250 °C under H_2.

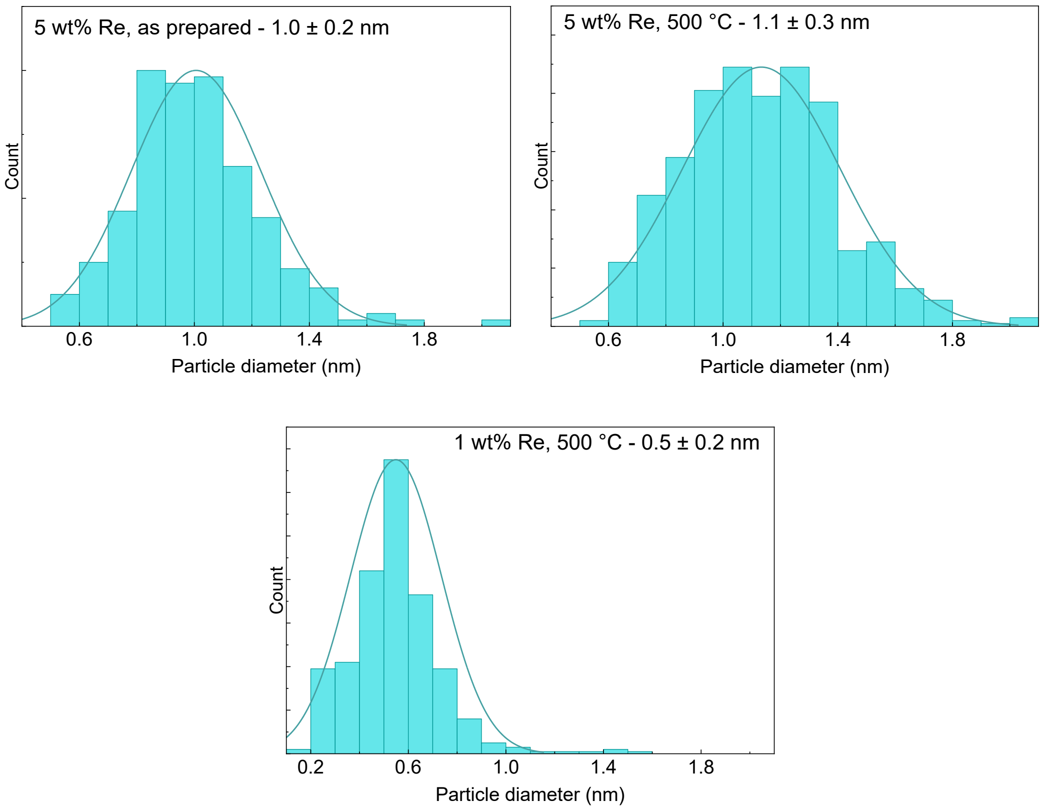

Figure S4. Particle size distribution of Re NPs on Re/TiO_2 evaluated through measurement of >200 particles seen by HR-STEM.

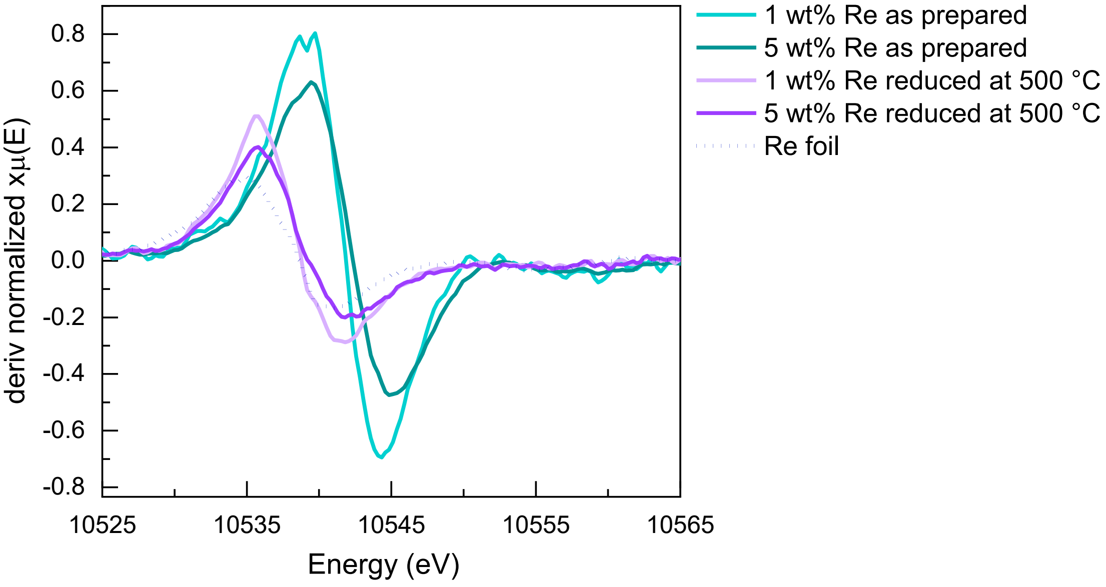

Figure S5. Normalized 1st derivative of Re L_3-edge XANES of Re/TiO_2 samples and Re foil standard.

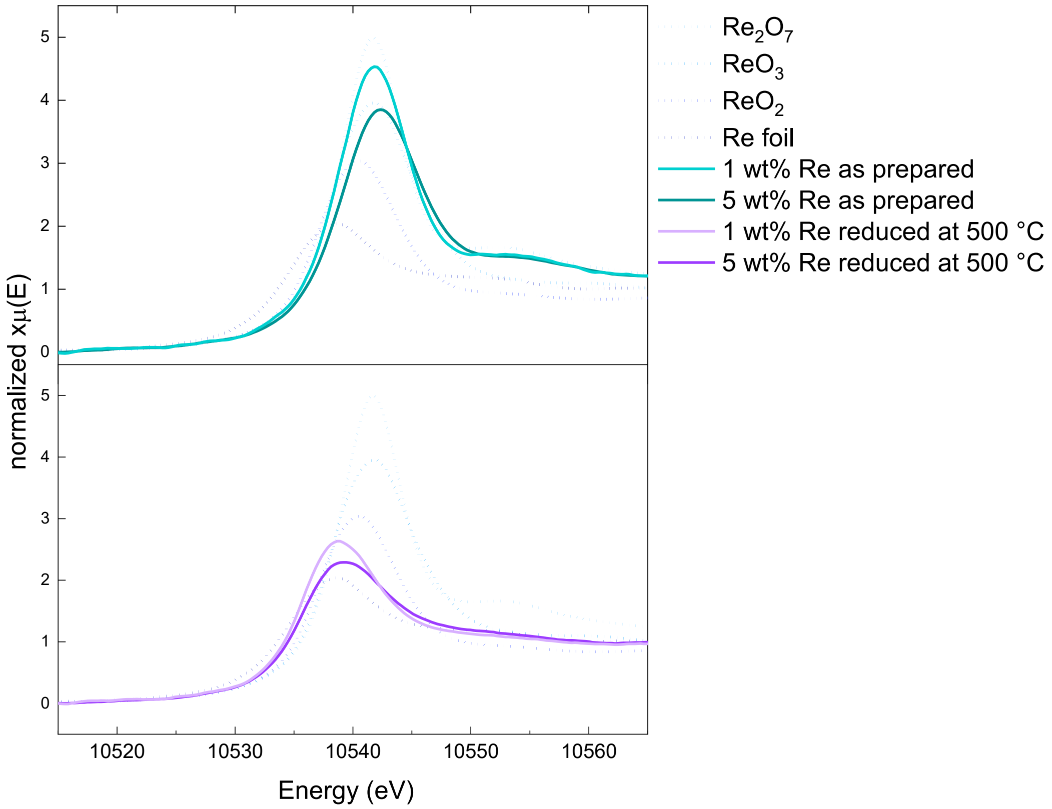

Figure S6. Re L_3-edge XANES comparison of Re/TiO_2 to Re standards.

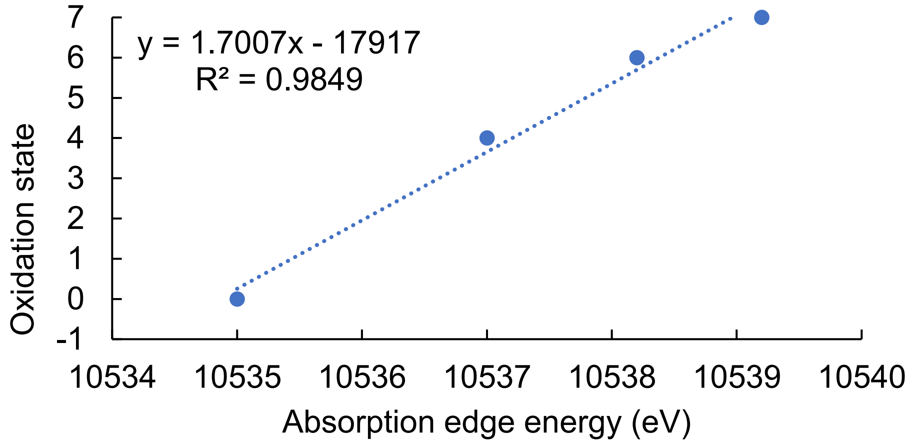

Figure S7. Oxidation state of Re as a function of absorption edge energy

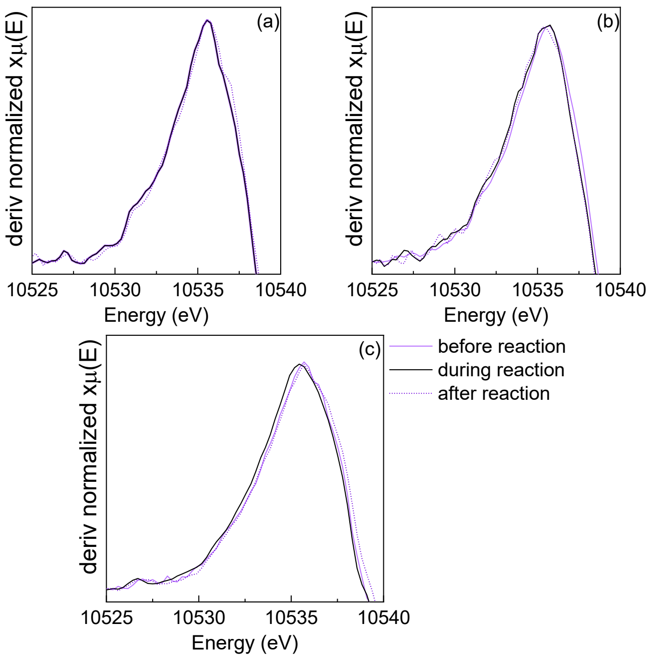

Figure S8. 1st derivative of μ(E) obtained from operando XANES of CO_2 hydrogenation reaction over Re/TiO_2: (a) 1 wt% Re, pre-reduced at 500 °C; (b) 5 wt% Re, pre-reduced at 250 °C; (c) 5 wt% Re, pre-reduced at 500 °C.
Reaction conditions: fixed-bed quartz reactor packed 70 mg of Re/TiO_2, P = 20 bar, CO_2:H_2 = 1:4, T = 200 °C, GHSV = 10,000 $mL.g_{cat}^{-1}.h^{-1}$.

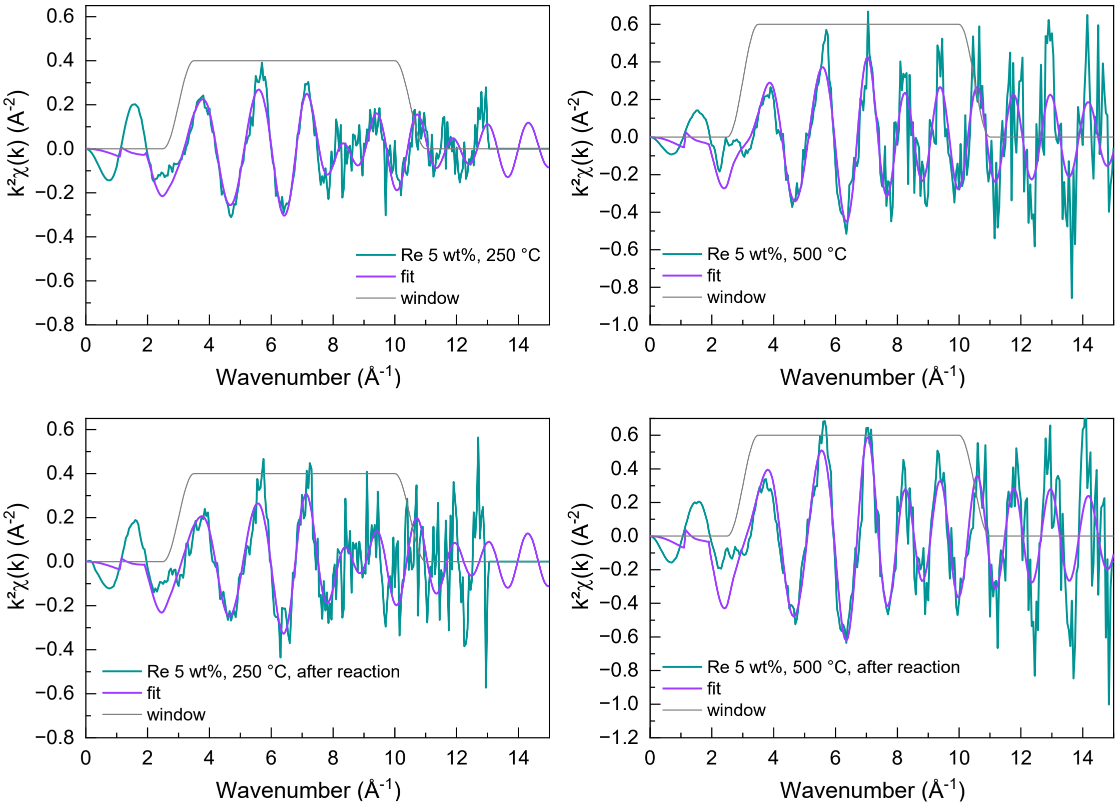

Figure S9. The k_2-weighted Re L_3-edge EXAFS in k space are shown for Re/TiO_2 with 5 wt% Re (top left) reduced at 250 °C, (top right) reduced at 500 °C, (bottom left) after reaction of 250°C reduced, (bottom right) after reaction of 500°C reduced. The data is shown in green, and the EXAFS best fit is shown in purple. The k-window of 3-11 Å^{-1} used for Fourier transform is also shown in black.

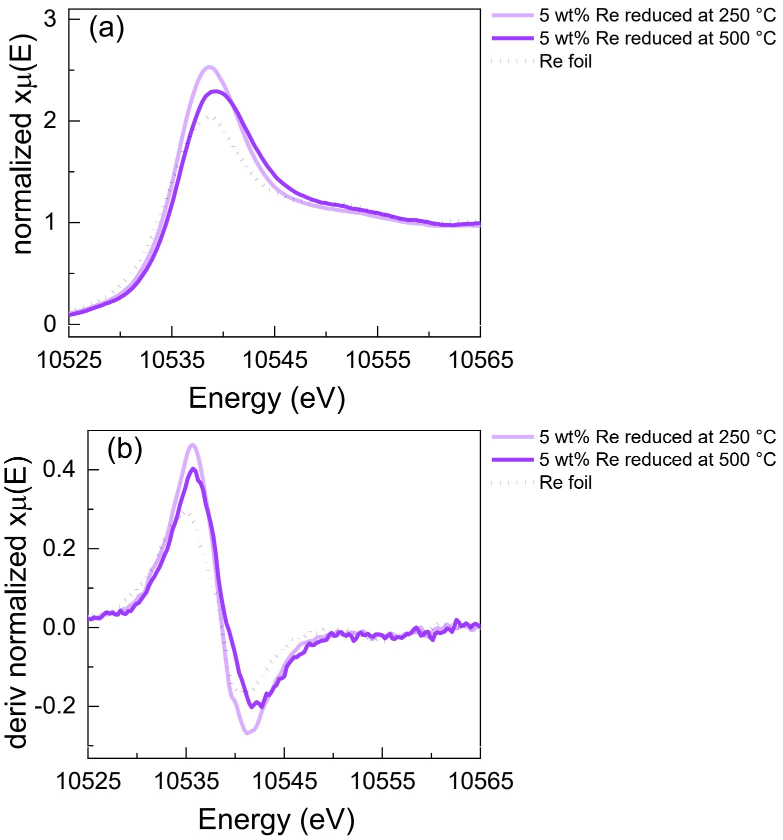

Figure S10. Room-temperature measurements of Re L_3-edge XANES of Re/TiO_2 reduced at 250 °C and 500 °C plotted in (a) normalized μ(E) and (b) normalized 1st derivative of μ(E).

Thermodynamic equilibrium

For the simulation performed in Aspen Plus (V. 8.8), the SRK EOS model with modifications by Mathias[1] was used. The well-known Soave-Redlich-Kwong equation of state is represented by Equation (SI 1).

$$P = \frac{RT}{V-b} - \frac{a(T)}{V(V+b)} \qquad (SI\ 1)$$

Mathias[1] proposed a change in the term $a(T)$, as seen in the Equations (Equations SI 2-13) sequence in Table S1. The change is precisely in the equation for $\alpha_i(T)$, which varies for pure fluids and supercritical substances. On the other hand, for mixtures, the terms a and b depend on the molar composition. This modification was necessary since SRK EOS generates data with a large error percentage when used for polar compounds like methanol[2]. The critical temperature ($T_{c,i}$), critical pressure ($P_{c,i}$), acentric factor (ω_i), and polarity correction factor (p_i) used are given in Table S2. The binary interaction parameter (k_{ij}) values are given in Table S3.

Table S1. The thermodynamic model used in simulations.

	For pure fluids
$\boldsymbol{a(T)}$	$a(T) = a_i \cdot \alpha_i(T) \qquad (SI\ 2)$
	$a_i = 0.42747 \dfrac{R^2 T_{c,i}^2}{P_{c,i}^2} \qquad (SI\ 3)$
	$\alpha_i^{0.5}(T) = 1 + m_i\left(1 - \sqrt{T_{R,i}}\right) - p_i\left(1 - T_{R,i}\right)\left(0.7 - T_{R,i}\right) \qquad (SI\ 4)$
	$T_{R,i} = \dfrac{T}{T_{c,i}} \qquad (SI\ 5)$
	$m_i = 0.48508 + 1.5519\omega_i - 0.15613\omega_i^2 \qquad (SI\ 6)$
$\boldsymbol{b}$	$b_i = 0.08664 \dfrac{R T_{c,i}}{P_{c,i}} \qquad (SI\ 7)$
	**For supercritical substances**
$\boldsymbol{a(T)}$	$\alpha_i^{0.5}(T) = e^{c_i\left(1 - T_{R,i}{}^{d_i}\right)} \qquad (SI\ 8)$
	$c_i = 1 + \dfrac{m_i}{2} + 0.3p_i \qquad (SI\ 9)$
	$d_i = \dfrac{c_i - 1}{c_i} \qquad (SI\ 10)$
	**For mixtures**
$\boldsymbol{a(T)}$	$a = \sum_{i=1}^{n} \sum_{j=1}^{n} y_i \cdot y_j \cdot a_{ij} \qquad (SI\ 11)$
	$a_{ij} = \sqrt{a_i a_j}\left(1 - k_{ij}\right) \qquad (SI\ 12)$
$\boldsymbol{b}$	$b = \sum_{i=1}^{n} y_i \cdot b_i \qquad (SI\ 13)$

Equations from Mathias[1], Bennekom et al.[2], and Perry et al.[3]

Table S2. Component parameters.

	$T_{c,i}$	$P_{c,i}$	ω_i	p_i
CO_2	304.12 K	7,374 kPa	0.225	0
H_2	32.98 K	1,293 kPa	-0.217	0
CH_3OH	512.64 K	8,097 kPa	0.565	0.2359
H_2O	647.14 K	22,064 kPa	0.344	0.1277
CO	132.85 K	3,494 kPa	0.045	0
CH_4	190.56 K	4,599 kPa	0.011	0

Data already reported by Bennekom et al.[2]

Table S3. Parameters for binary interactions (k_{ij}) for the modified SRK EOS.

Component (i)	Component (j)	$k_{ij} = k_{ji}$
CO_2	H_2	0.1164
CO_2	CH_3OH	0.1000
CO_2	H_2O	0.3000
CO_2	CO	0.1164
CO_2	CH_4	0.0956
H_2	CH_3OH	-0.1250
H_2	H_2O	-0.7450
H_2	CO	-0.0007
H_2	CH_4	0.0010
CH_3OH	H_2O	-0.0750
CH_3OH	CO	-0.3700
CH_3OH	CH_4	0.0460
H_2O	CO	-0.4740
H_2O	CH_4	0.0140
CO	CH_4	0.0204

Data already reported by Bennekom et al.[2]

Table S4 shows the vapor fraction and other information for the inlet and outlet streams of the results obtained from the simulation for a 1:4 molar ratio (CO_2:H_2) reaction. Note that in all scenarios, the vapor fraction is 1, i.e., 100% of the streams are in the gaseous state despite the different compositions in the RGibbs module outlet streams. All reaction mixtures for the other reactant molar ratios also resulted in a 100% vapor fraction.

The thermodynamic equilibrium, already discussed in the main text, is described in terms of CO_2 conversion in Figure S19 (a). However, we also decided to evaluate the scenario without the CH_4 compound since the Re/TiO_2 catalyst does not tend to produce it in representative quantities. Figure S19 (b) presents the CO_2 conversion of this scenario, and it is possible to see that the conversion increases with increasing pressure. However, it varies to a greater extent from 7% to 100%.

Table S4. Vapor fraction for inlet (ratio 1:4 CO_2:H_2) and outlet streams under different simulated conditions using RGibbs module.

$T\ (°C)$	$P\ (bar)$	CO_2 $conv$ (%)	V_f_{in}	$y_{CO_2,out}$	$y_{H_2,out}$	$y_{CH_3OH,out}$	$y_{H_2O,out}$	$y_{CO,out}$	$y_{CH_4,out}$	V_f_{out}	$T_C\ (°C)$	$P_C\ (bar)$
150	1	99.6080	1	0.00130	0.00521	0.00E+00	0.66232	1.29E-08	0.33116	1	219.11	161.53
150	20	99.8930	1	0.00036	0.00143	0.00E+00	0.66548	1.08E-09	0.33274	1	221.04	162.18
150	40	99.9286	1	0.00024	0.00095	0.00E+00	0.66587	5.54E-10	0.33294	1	221.28	162.26
150	60	99.9485	1	0.00017	0.00069	0.00E+00	0.66610	3.45E-10	0.33305	1	221.42	162.31
150	80	99.9647	1	0.00012	0.00047	1.35E-12	0.66627	2.14E-10	0.33314	1	221.53	162.34
150	100	99.9781	1	0.00007	0.00029	1.88E-12	0.66642	1.24E-10	0.33321	1	221.62	162.37
150	120	99.9806	1	0.00006	0.00026	1.97E-12	0.66645	1.07E-10	0.33323	1	221.64	162.38
200	1	98.8996	1	0.00364	0.01457	1.64E-12	0.65453	3.41E-07	0.32726	1	214.35	153.93
200	20	99.6907	1	0.00103	0.00412	5.84E-12	0.66324	2.92E-08	0.33162	1	219.67	161.72
200	40	99.7848	1	0.00072	0.00286	8.19E-12	0.66428	1.56E-08	0.33214	1	220.31	161.93
200	60	99.8341	1	0.00055	0.00221	1.03E-11	0.66482	1.03E-08	0.33241	1	220.64	162.04
200	80	99.8681	1	0.00044	0.00176	1.25E-11	0.66520	7.41E-09	0.33260	1	220.87	162.12
200	100	99.8952	1	0.00035	0.00140	1.50E-11	0.66550	5.44E-09	0.33275	1	221.06	162.18
200	120	99.9193	1	0.00027	0.00108	1.83E-11	0.66577	3.94E-09	0.33289	1	221.22	162.24
250	1	97.4441	1	0.00838	0.03352	1.15E-11	0.63873	4.85E-06	0.31936	1	204.69	156.68
250	20	99.2652	1	0.00244	0.00975	4.14E-11	0.65854	4.24E-07	0.32927	1	216.80	160.75
250	40	99.4762	1	0.00174	0.00696	5.70E-11	0.66087	2.31E-07	0.33043	1	218.22	161.23
250	60	99.5832	1	0.00139	0.00554	7.02E-11	0.66205	1.58E-07	0.33102	1	218.94	161.47
250	80	99.6543	1	0.00115	0.00460	8.25E-11	0.66283	1.18E-07	0.33142	1	219.43	161.63
250	100	99.7077	1	0.00097	0.00389	9.48E-11	0.66343	9.18E-08	0.33171	1	219.79	161.76
250	120	99.7509	1	0.00083	0.00332	1.08E-10	0.66390	7.34E-08	0.33195	1	220.08	161.85
300	1	94.8627	1	0.01656	0.06635	5.57E-11	0.61138	4.36E-05	0.30567	1	187.96	151.05
300	20	98.4921	1	0.00498	0.01992	2.06E-10	0.65007	3.88E-06	0.32503	1	211.62	159.01
300	40	98.9072	1	0.00362	0.01447	2.82E-10	0.65461	2.15E-06	0.32730	1	214.40	159.94
300	60	99.1140	1	0.00294	0.01175	3.43E-10	0.65688	1.49E-06	0.32844	1	215.78	160.41
300	80	99.2490	1	0.00249	0.00997	3.98E-10	0.65836	1.13E-06	0.32918	1	216.69	160.71
300	100	99.3484	1	0.00216	0.00865	4.49E-10	0.65946	9.06E-07	0.32973	1	217.36	160.94
300	120	99.4269	1	0.00190	0.00761	4.99E-10	0.66032	7.46E-07	0.33016	1	217.89	161.12

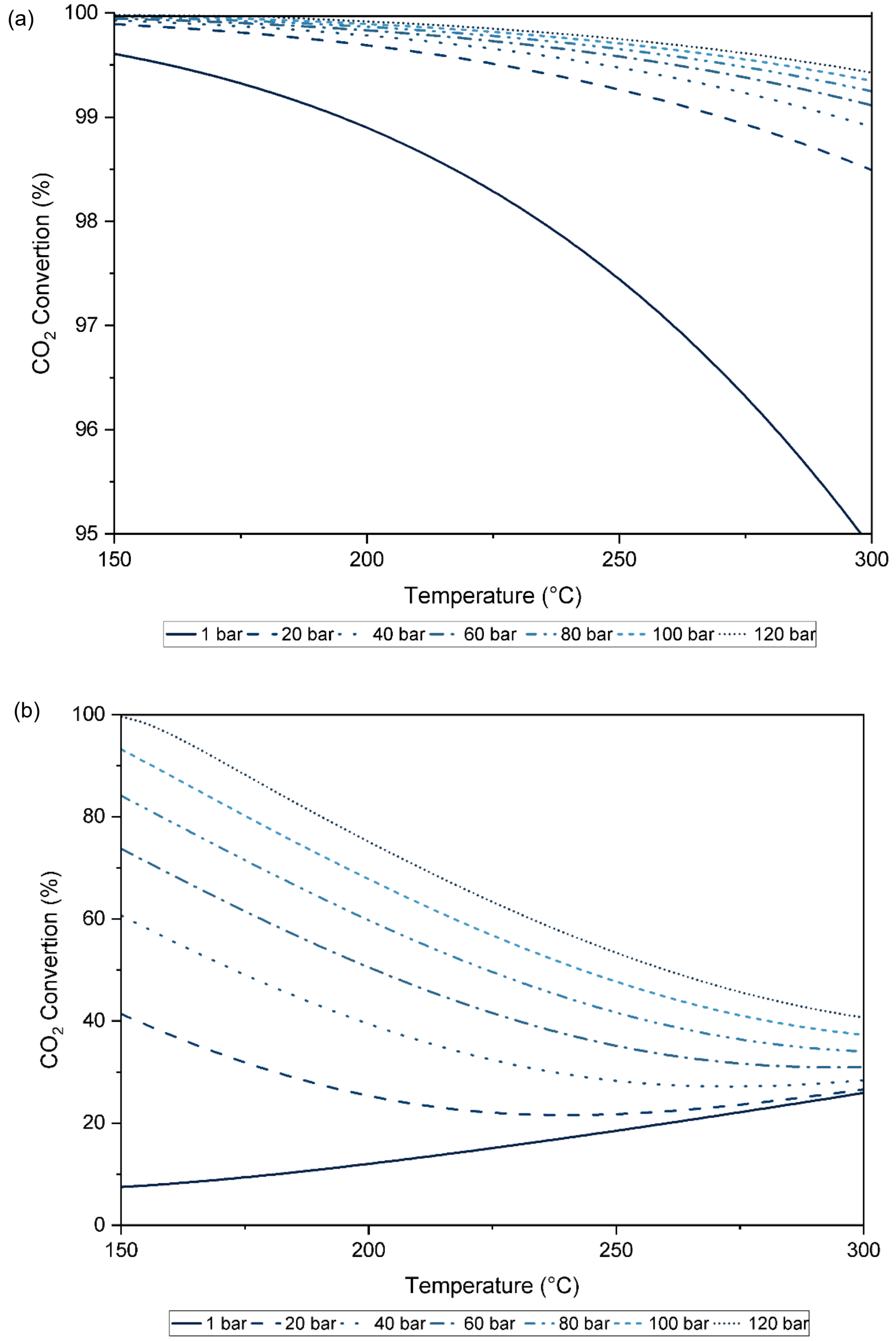

Figure S11. Thermodynamic equilibrium simulated for calculating CO_2 convertion (%) using ratio 1:4 (CO_2:H_2) (a) considering CO_2, H_2, CH_3OH, CO, and CH_4 (b) considering CO_2, H_2, CH_3OH, and CO.

From SI Tables 5 and 6, it is possible to conclude that the molar ratio greatly influences CO_2 conversion. However, neither temperature nor pressure interfere effectively in these scenarios for thermodynamic equilibrium.

Figure S23 shows the selectivity values already discussed in the main text in the 1:4 ratio scenario. In this Figure, with the calculation of the logarithm in base 10, it is possible to directly compare the selectivity of CH_3OH, CH_4, and CO. The same comparison was made for the other molar ratio: Figure S20 1:1; Figure S21 1:2; Figure S22 1:3; Figure S23 1:4; Figure S24 1:5. In all molar ratio scenarios, the selectivity of CH_4 was almost 100%, that is, close to 2 since the Gibbs energy minimization method results in greater selectivity for the more stable compound. Note that for CH_3OH and CO, the values are always very low, which decreases as the amount of H_2 in the reaction medium increases. This reflects the tendency for more CH_4 to be formed at thermodynamic equilibrium.

Table S5. Thermodynamic equilibrium simulated for calculating CO_2 convertion versus temperature using different ratios CO_2:H_2 (1:1, 1:2, 1:3, 1:4, 1:5).

T (°C)	1:1	1:2	1:3	1:4	1:5
150	24.99899	49.99878	74.99782	99.98055	100
170	24.99793	49.99644	74.99571	99.97133	100
190	24.99618	49.99282	74.98877	99.94146	100
210	24.99343	49.98707	74.97861	99.89447	100
230	24.98936	49.97833	74.96311	99.83282	100
250	24.98358	49.96558	74.94027	99.75089	100
270	24.97574	49.94766	74.90777	99.64405	100
290	24.96555	49.9233	74.86299	99.50766	100
300	24.95952	49.90829	74.83508	99.4269	100

Table S6. Thermodynamic equilibrium simulated for calculating CO2 convertion versus pressure using different ratios CO_2:H_2 (1:1, 1:2, 1:3, 1:4, 1:5).

P (bar)	1:1	1:2	1:3	1:4	1:5
1	24.90579	49.79512	74.63036	98.89957	99.99999
20	24.98041	49.95773	74.92397	99.69066	100
40	24.98724	49.97279	74.95144	99.78483	100
60	24.99045	49.97994	74.96461	99.83407	100
80	24.99246	49.98447	74.97306	99.86809	100
100	24.99387	49.98774	74.97928	99.89516	100
120	24.99495	49.99026	74.98424	99.91928	100

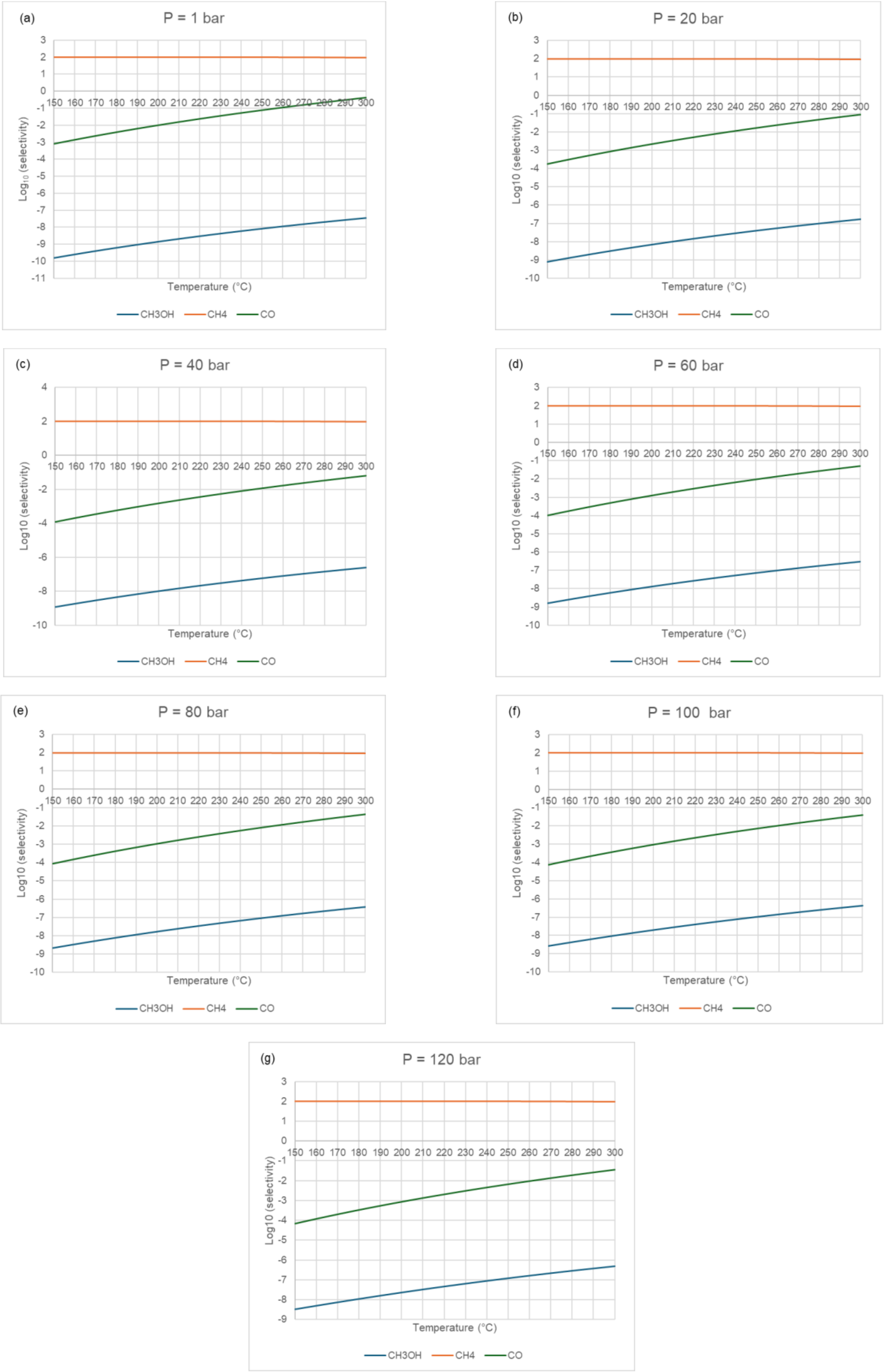

Figure S12. Log_{10} of Selectivity (%) at thermodynamic equilibrium for CH_3OH, CH_4, and CO using ratio 1:1 (CO_2:H_2) at different pressures (a) 1 bar (b) 20 bar (c) 40 bar (d) 60 bar (e) 80 bar (f) 100 bar (g) 120 bar.

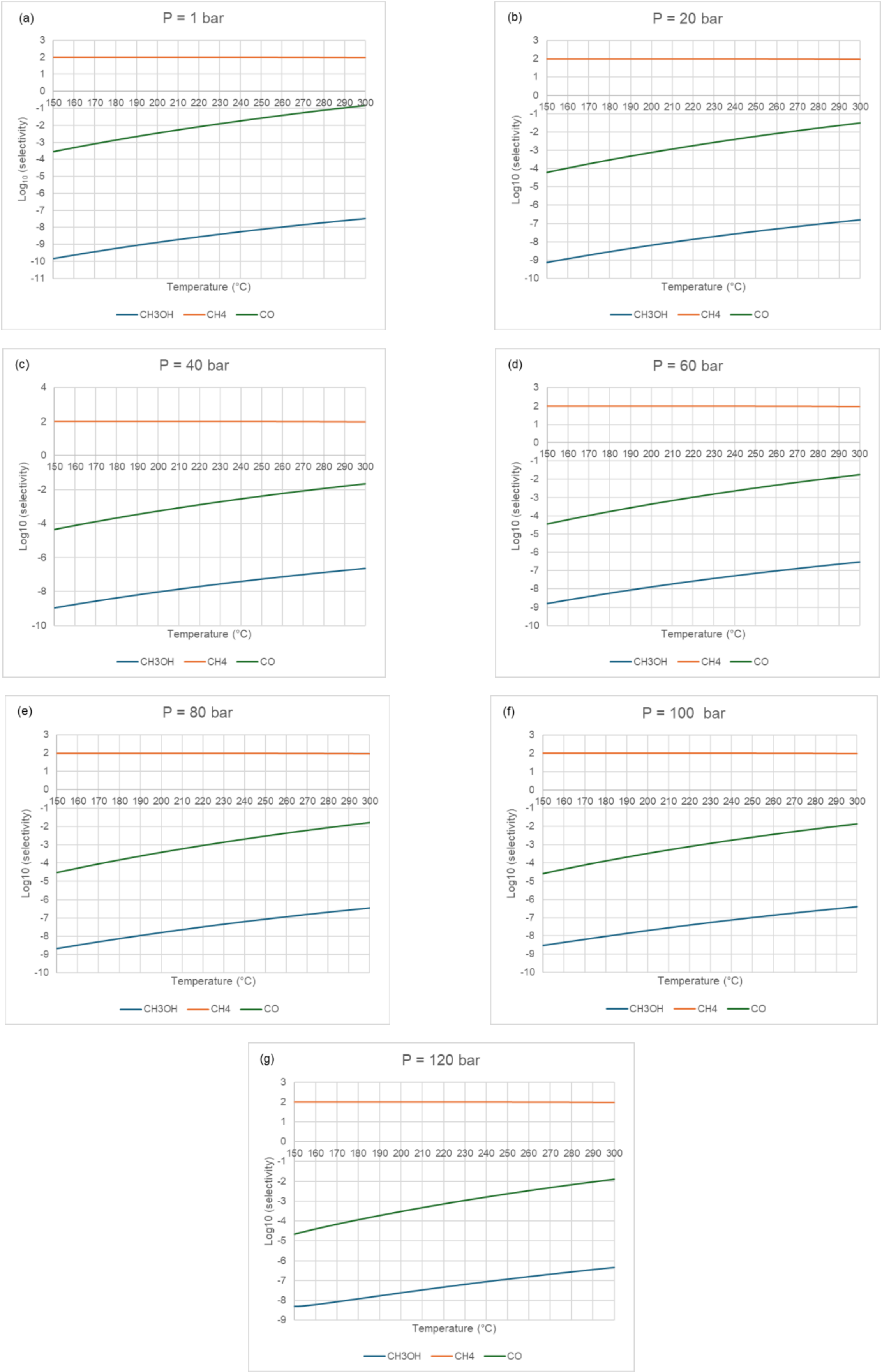

Figure S13. Log_{10} of Selectivity (%) at thermodynamic equilibrium for CH_3OH, CH_4, and CO using ratio 1:2 (CO_2:H_2) at different pressures (a) 1 bar (b) 20 bar (c) 40 bar (d) 60 bar (e) 80 bar (f) 100 bar (g) 120 bar.

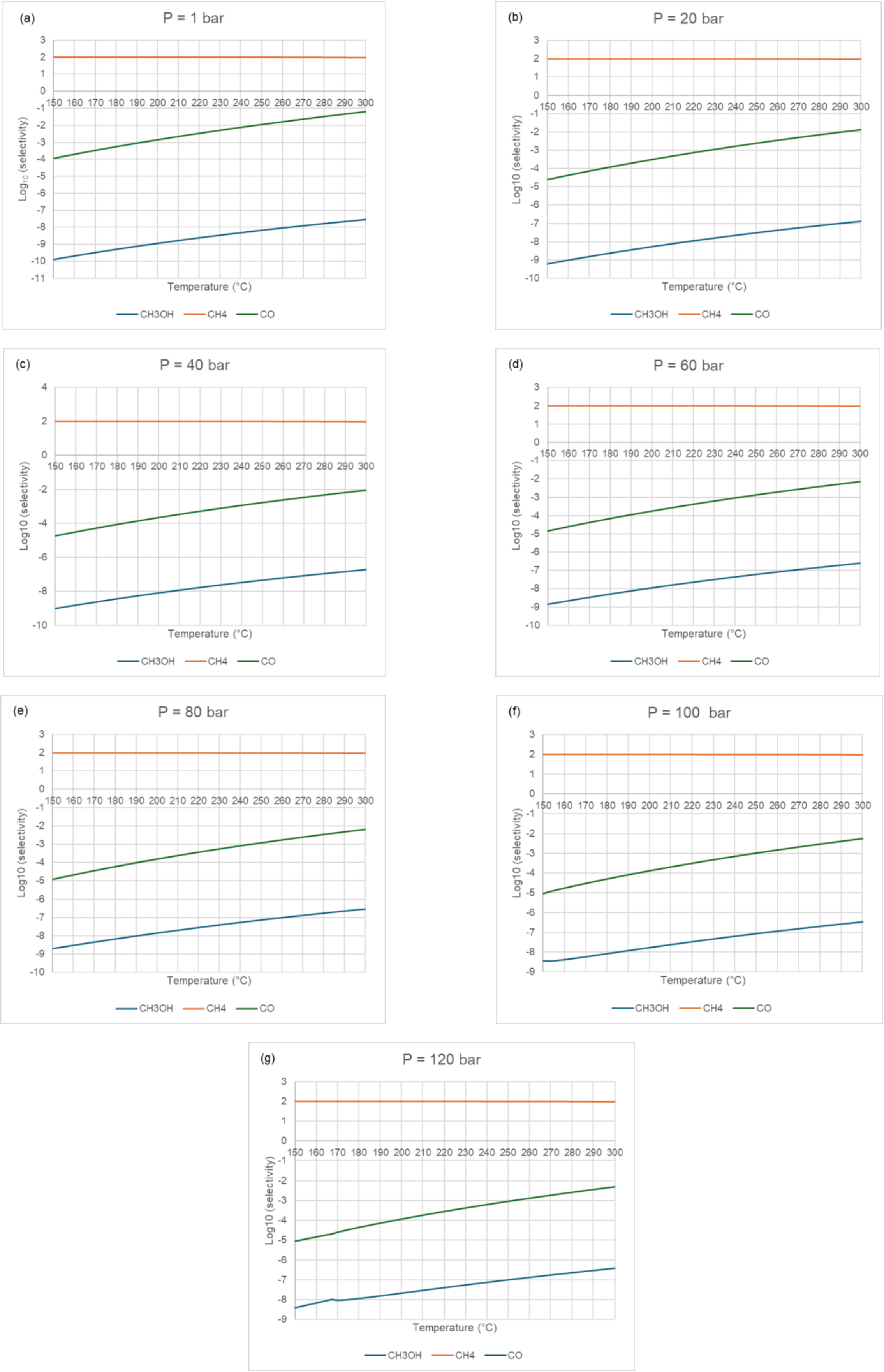

Figure S14. Log_{10} of Selectivity (%) at thermodynamic equilibrium for CH_3OH, CH_4, and CO using ratio 1:3 (CO_2:H_2) at different pressures (a) 1 bar (b) 20 bar (c) 40 bar (d) 60 bar (e) 80 bar (f) 100 bar (g) 120 bar.

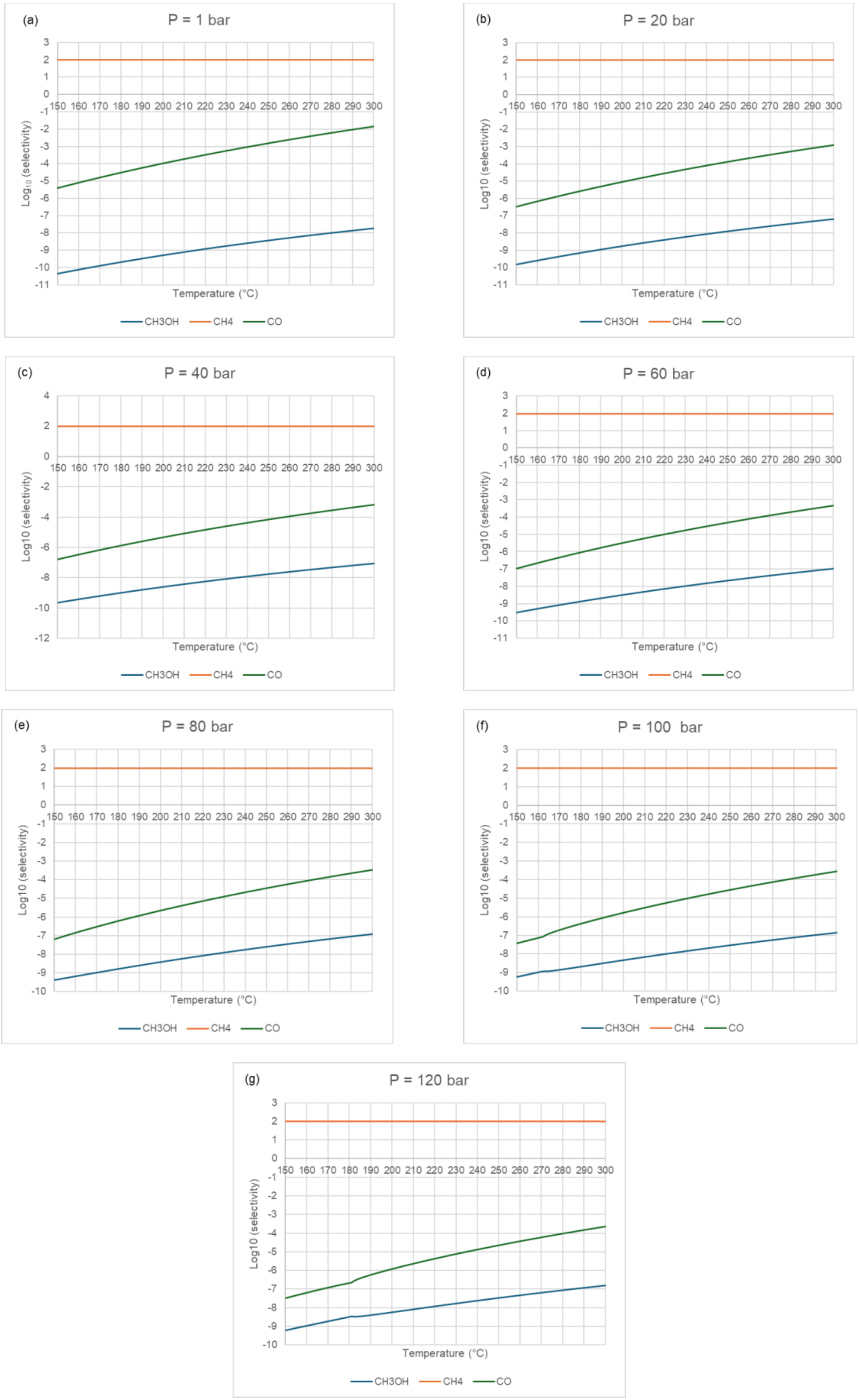

Figure S15. Log_{10} of Selectivity (%) at thermodynamic equilibrium for CH_3OH, CH_4, and CO using ratio 1:4 (CO_2:H_2) at different pressures (a) 1 bar (b) 20 bar (c) 40 bar (d) 60 bar (e) 80 bar (f) 100 bar (g) 120 bar.

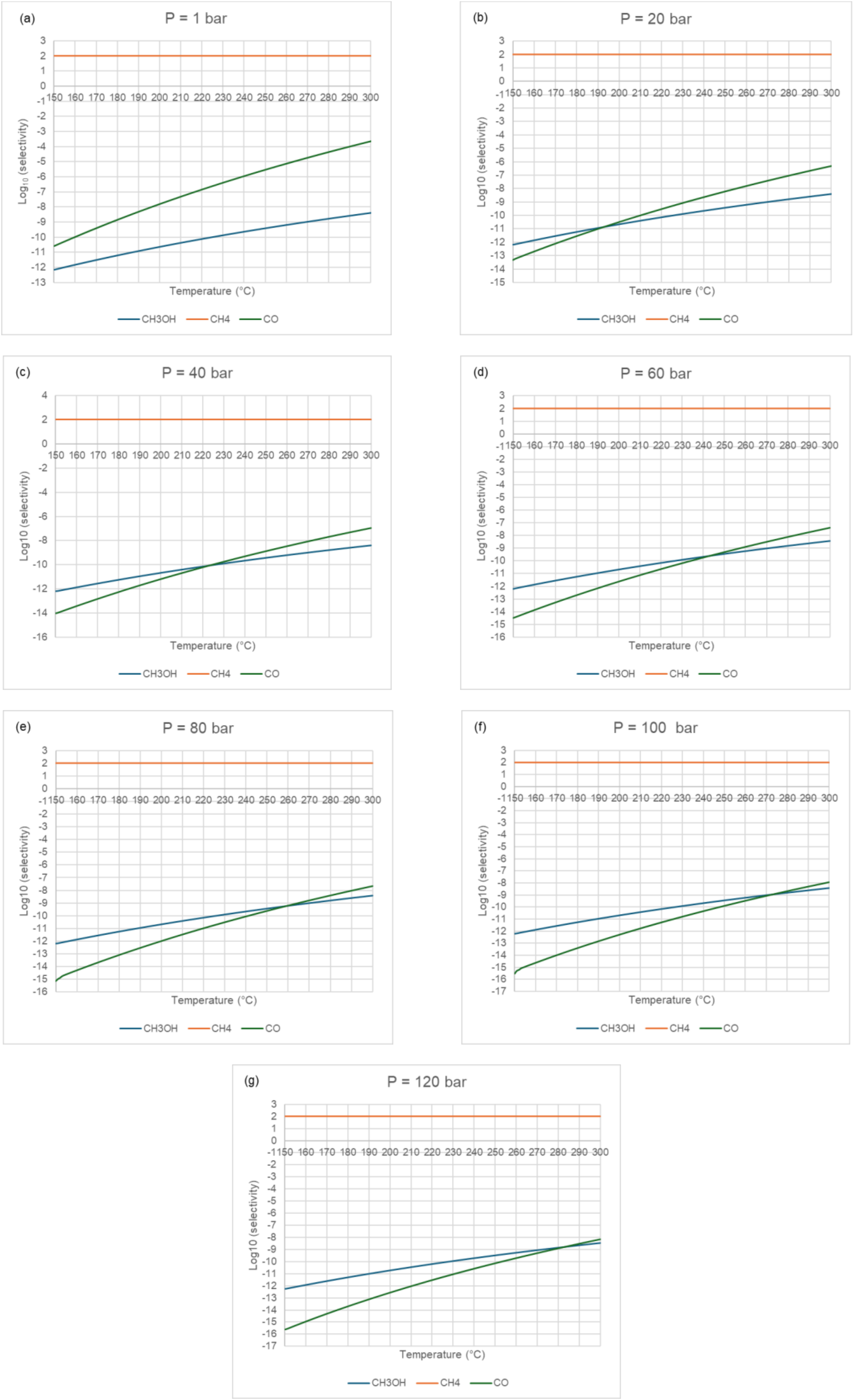

Figure S16. Log_{10} of Selectivity (%) at thermodynamic equilibrium for CH_3OH, CH_4, and CO using ratio 1:5 (CO_2:H_2) at different pressures (a) 1 bar (b) 20 bar (c) 40 bar (d) 60 bar (e) 80 bar (f) 100 bar (g) 120 bar.

Density functional theory calculations

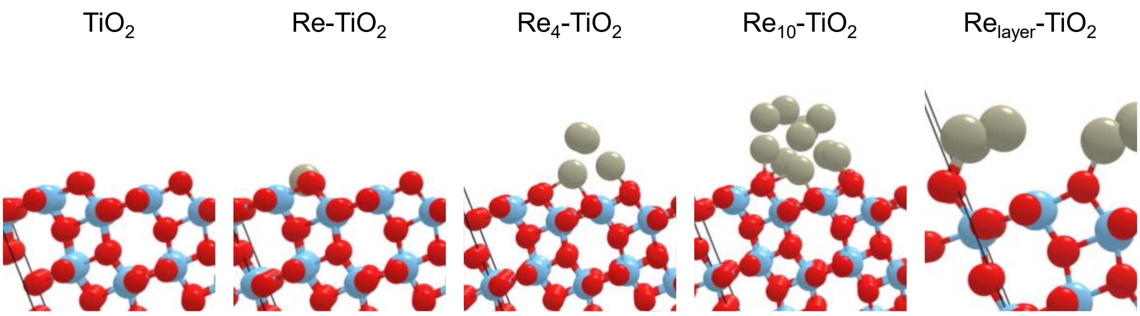

Figure S17. Side view of theoretical models studied in this work.

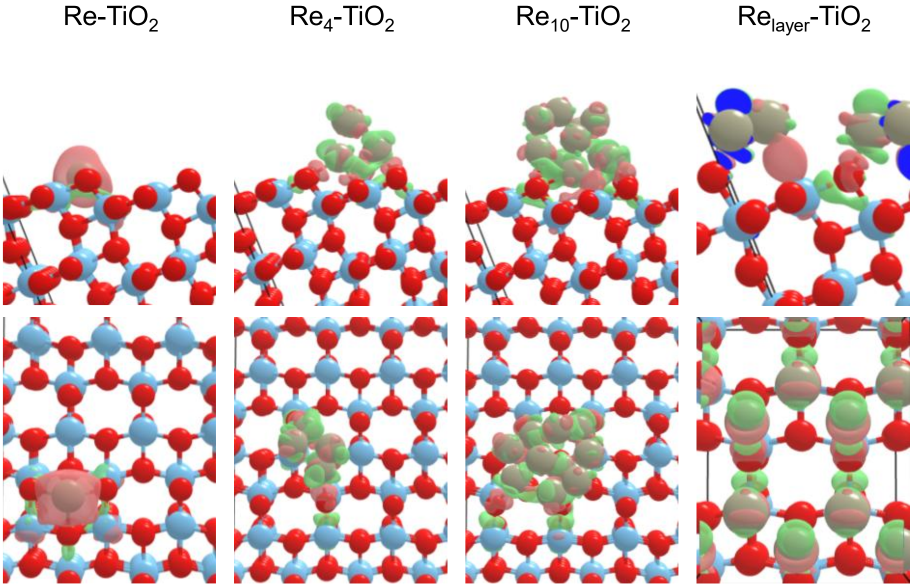

Figure S18. Top and side view charge density difference for the interaction between Re, Re_4, Re_{10}, and Re_{layer} with TiO_2. Green indicates negative charge density, and red indicates positive charge density.

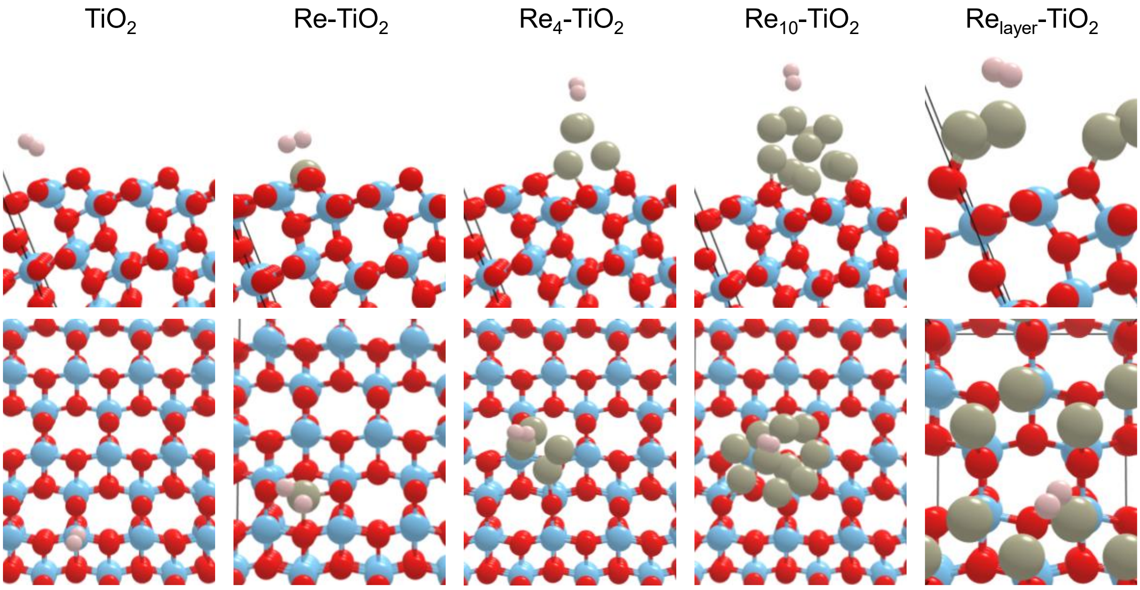

Figure S19. Top and side view of H_2 molecule adsorption in the theoretical models studied in this work.

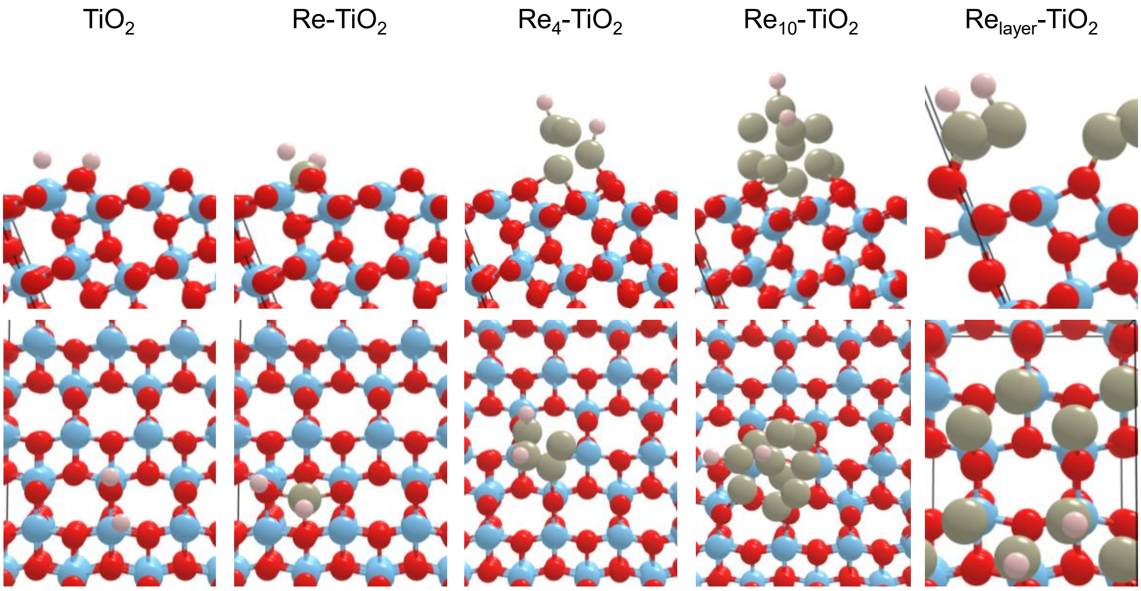

Figure S20. The top and side views of H_2 molecules after dissociation in the theoretical models studied in this work.

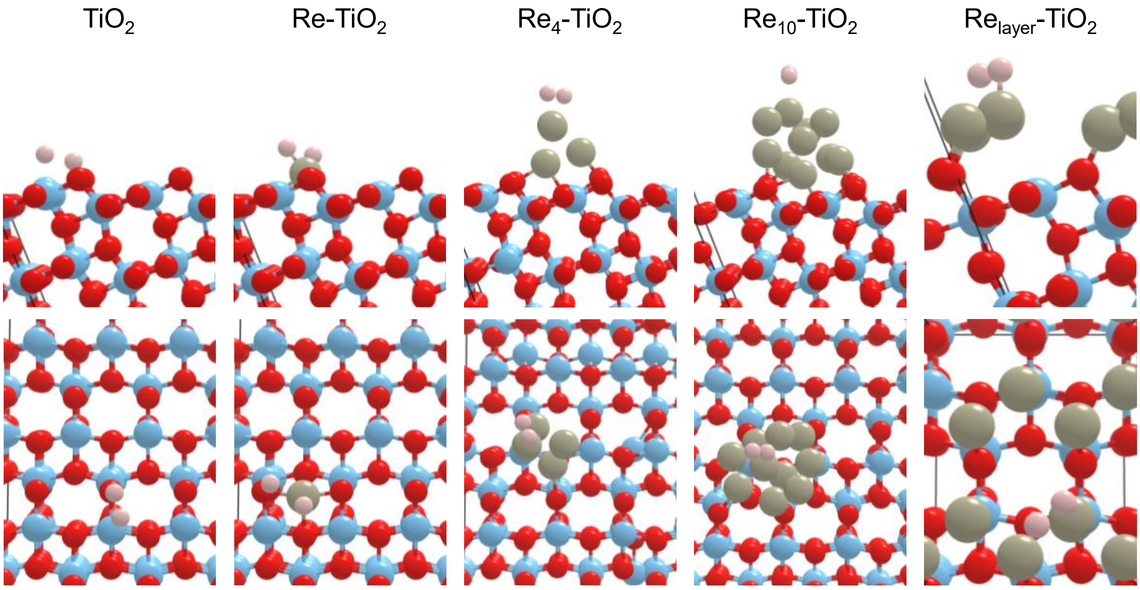

Figure S21. Top and side view of TS state for H_2 molecule dissociation in the theoretical models studied in this work.

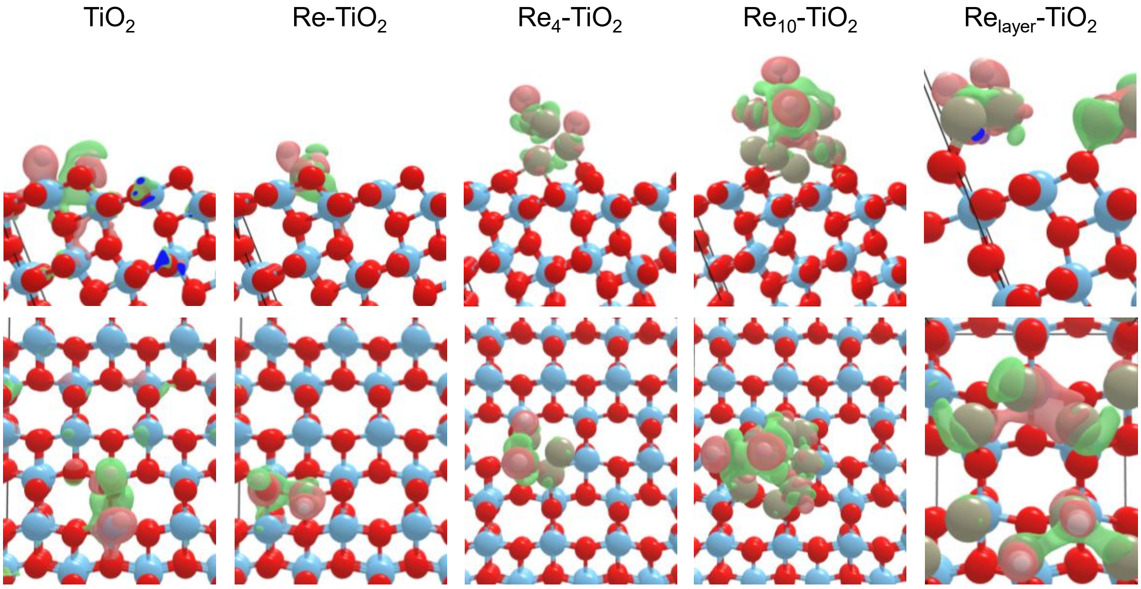

Figure S22. Top and side view charge density difference for H_2 molecule after dissociation in the theoretical models studied in this work. Green indicates negative charge density, and red indicates positive charge density.

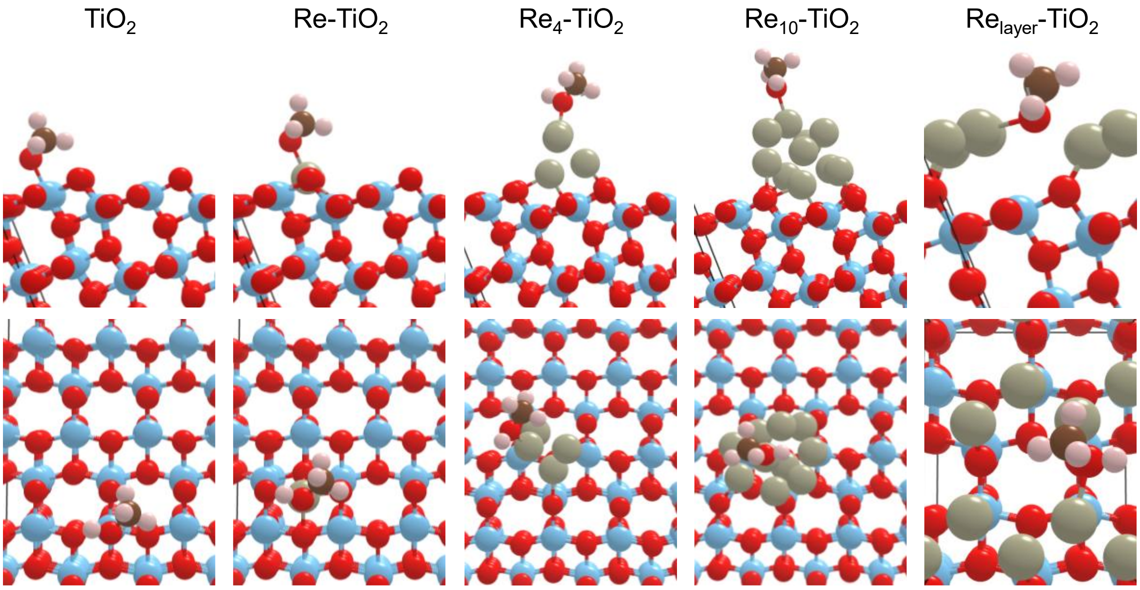

Figure S23. Top and side view of CH_3OH molecule adsorption in the theoretical models studied in this work.

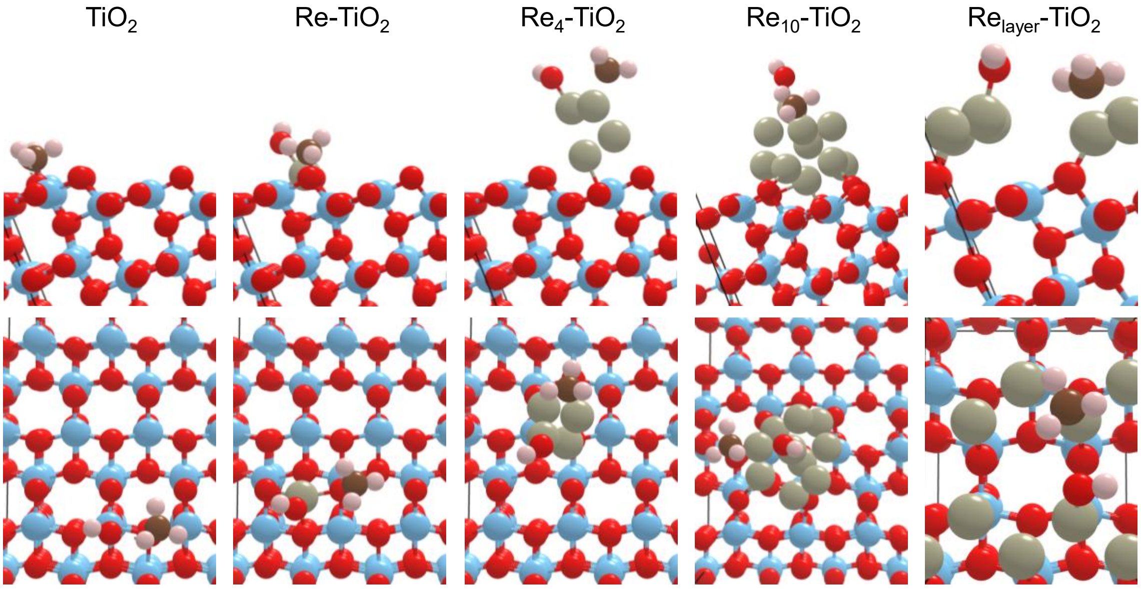

Figure S24. The top and side views of the CH_3OH molecule after dissociation in the theoretical models studied in this work.

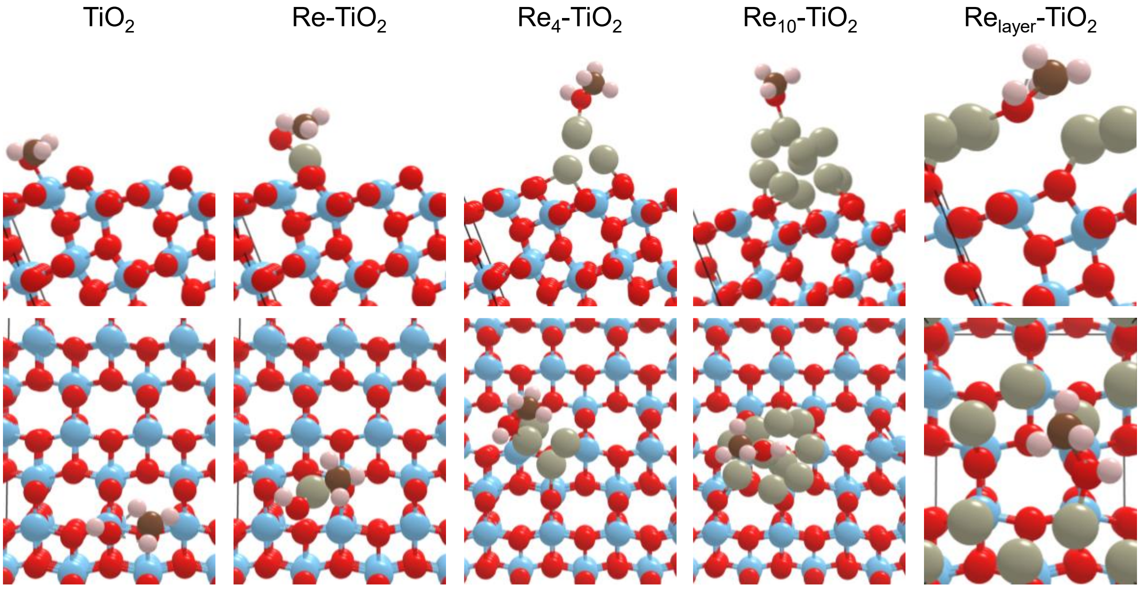

Figure S25. Top and side view of TS state for CH_3OH molecule dissociation in the theoretical models studied in this work.

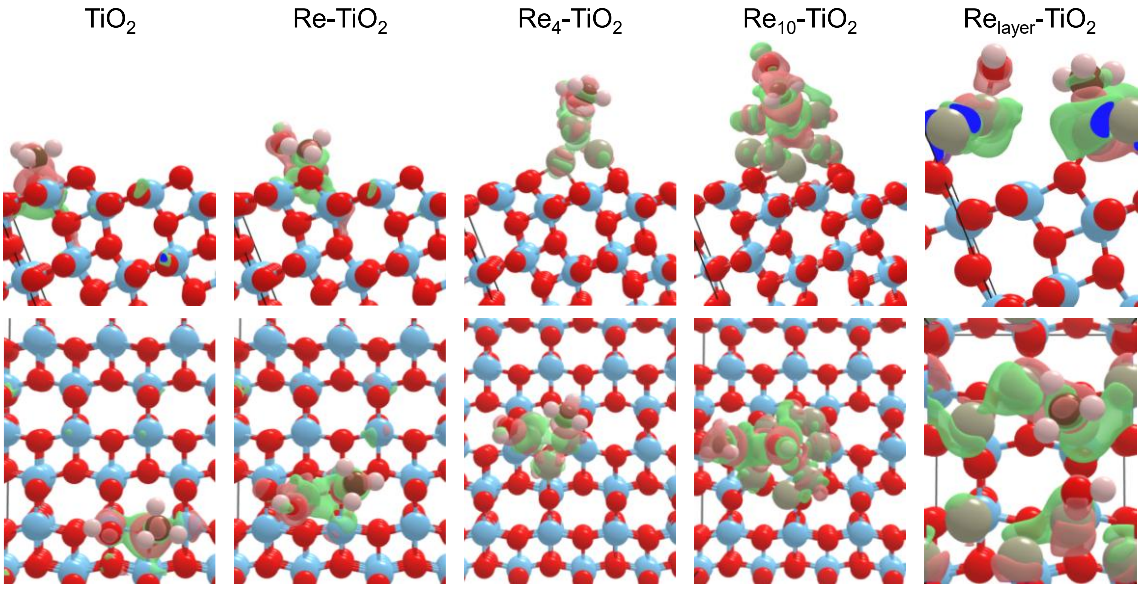

Figure S26. Top and side view charge density difference for CH_3OH molecule after dissociation in the theoretical models studied in this work. Green indicates negative charge density, and red indicates positive charge density.

SI References

(1) Mathias, P. M. A Versatile Phase Equilibrium Equation of State. Industrial & Engineering Chemistry Process Design and Development 1983, 22 (3), 385–391. https://doi.org/10.1021/i200022a008

(2) Bennekom, J. G. van; Winkelman, J. G. M.; Venderbosch, R. H.; Nieland, S. D. G. B.; Heeres, H. J. Modeling and Experimental Studies on Phase and Chemical Equilibria in High-Pressure Methanol Synthesis. Industrial & Engineering Chemistry Research 2012, 51 (38), 12233–12243. https://doi.org/10.1021/ie3017362.

(3) Perry, R. H.; Green, D. W.; Maloney, J. O. Perry's Chemical Engineers' Handbook; Science Pr: Beijing, China, 2001.
